# Simulating Transmission Scenarios of the Delta Variant of SARS-CoV-2 in Australia

**DOI:** 10.3389/fpubh.2022.823043

**Published:** 2022-02-24

**Authors:** Sheryl L. Chang, Oliver M. Cliff, Cameron Zachreson, Mikhail Prokopenko

**Affiliations:** ^1^Centre for Complex Systems, Faculty of Engineering, The University of Sydney, Sydney, NSW, Australia; ^2^School of Physics, The University of Sydney, Sydney, NSW, Australia; ^3^School of Computing and Information Systems, The University of Melbourne, Parkville, VIC, Australia; ^4^Sydney Institute for Infectious Diseases, The University of Sydney, Westmead, NSW, Australia

**Keywords:** COVID-19, SARS-CoV-2, Delta (B.1.617.2) variant, computational epidemiology, agent-based model, social distancing, vaccination, healthcare burden

## Abstract

An outbreak of the Delta (B.1.617.2) variant of SARS-CoV-2 that began around mid-June 2021 in Sydney, Australia, quickly developed into a nation-wide epidemic. The ongoing epidemic is of major concern as the Delta variant is more infectious than previous variants that circulated in Australia in 2020. Using a re-calibrated agent-based model, we explored a feasible range of non-pharmaceutical interventions, including case isolation, home quarantine, school closures, and stay-at-home restrictions (i.e., “social distancing.”) Our modelling indicated that the levels of reduced interactions in workplaces and across communities attained in Sydney and other parts of the nation were inadequate for controlling the outbreak. A counter-factual analysis suggested that if 70% of the population followed tight stay-at-home restrictions, then at least 45 days would have been needed for new daily cases to fall from their peak to below ten per day. Our model predicted that, under a progressive vaccination rollout, if 40–50% of the Australian population follow stay-at-home restrictions, the incidence will peak by mid-October 2021: the peak in incidence across the nation was indeed observed in mid-October. We also quantified an expected burden on the healthcare system and potential fatalities across Australia.

## 1. Introduction

Strict mitigation and suppression measures eliminated local transmission of SARS-CoV-2 during the initial pandemic wave in Australia (March–June 2020; peaked around 500 cases per day, i.e., around 20 daily cases per million) (1), as well as a second wave that developed in the south-eastern state of Victoria (June–September 2020; peaked around 700 cases per day, i.e., around 30 daily cases per million) (2, 3)^1^. Several subsequent outbreaks were also detected and managed quickly and efficiently by contact tracing and local lockdowns, e.g., a cluster in the Northern Beaches Council of Sydney, New South Wales (NSW) totalled 217 cases and was controlled in 32 days by locking down only the immediately affected suburbs (December 2020–January 2021) (5). Overall, successful pandemic response was facilitated by effective travel restrictions and stringent stay-at-home restrictions (i.e., “social distancing,”) underpinned by a high-intensity disease surveillance (6–10).

Unfortunately, the situation changed in mid-June 2021, when a highly transmissible variant of concern, B.1.617.2 (Delta), was detected. The first infection was recorded on June 16 in Sydney, and quickly spread through the Greater Sydney area. Within ten days, there were more than 100 locally acquired cumulative cases, triggering stay-at-home (social distancing) restrictions imposed in Greater Sydney and nearby areas (11). By July 9 (23 days later), the locally acquired cases reached 439 in total (5), and a tighter lockdown was announced (11). Further restrictions and business shut-downs, including construction and retail industries, were announced on 17 July (12). By then, the risk of a prolonged lockdown had become apparent (13), with the epidemic spreading to the other states and territories, most notably Victoria (VIC) and the Australian Capital Territory (ACT). The incidence peaked, around 2,750 daily cases, i.e., around 100 daily cases per million, only by mid-October 2021, and stabilised in November within the range between 1,200 and 1,600 daily cases, i.e., between 45 and 65 daily cases per million (5), before a new surge of infections in December 2021 due to the Omicron variant (B.1.1.529).

The difficulty of controlling the third epidemic wave (June–November 2021) is attributed to a high transmissibility of the B.1.617.2 (Delta) variant, which is known to increase the risk of household transmission by approximately 60% in comparison to the B.1.1.7 (Alpha) variant (14). This transmissibility was compounded by the initially low rate of vaccination in Australia, with around 6% of the adult population double vaccinated before the Sydney outbreak and only 7.92% of adult Australians double vaccinated by the end of June 2021 (15), with this fraction increasing to 67.24% by 15 October 2021 and 83.01% by 13 November 2021 (16).

Several additional factors make the Sydney outbreak and the third pandemic wave in Australia (June–November 2021) an important case study, in which the system complexity and the search space formed by possible interventions can be reduced. Because previous pandemic waves were eliminated in Australia, the Delta variant has not been competing with other variants. Secondly, the level of acquired immunity to SARS-CoV-2 in the Australian population was low at the onset of the outbreak, given that (a) the pre-existing natural immunity was limited by cumulative confirmed cases of around 0.12%, and (b) immunity acquired due to vaccination did not extend beyond 6% of the adult population. Furthermore, the school winter break in NSW (28 June–9 July) coincided with the period of social distancing restrictions announced on 26 June, with school premises remaining mostly closed beyond 9 July. Thus, the epidemic suppression policy of school closures is not a free variable, further reducing the search space of available control measures.

This study addresses several important questions. Firstly, we investigate a feasible range of key non-pharmaceutical interventions (NPIs): case isolation, home quarantine, school closures, and social distancing, available to control virus transmission within a population with a low immunity. Social distancing (SD) is interpreted and modelled in a broad sense of comprehensive stay-at-home restrictions, comprising several specific behavioural changes that reduce the intensity of interactions among individuals (and hence the virus transmission probability), including physical distancing, mobility reduction, mask wearing, and so on. Our primary focus is a “retrodictive” estimation of the average (unknown) SD level under which the modelled transmission and suppression dynamics can be best matched to the observed incidence data. An identification of the SD level helps to distinguish and evaluate the distinct and time-varying impacts of NPIs and vaccination campaigns.

Secondly, in a counter-factual mode, we quantify under what conditions the initial outbreak could have been suppressed, aiming to clarify the extent of required NPIs during an early outbreak phase with low vaccination coverage, in comparison to previous pandemic control measures successfully deployed in Australia. This analysis highlights the challenges associated with imposing very tight restrictions which would be required to suppress the high transmissible Delta variant.

Finally, we offer and validate a projection for the peak of case incidence across the nation, formed in response to a progressive vaccination campaign rolling out concurrently with the strict lockdown measures adopted in NSW, VIC, and ACT. In doing so, we predict the expected hospitalisations, intensive care unit (ICU) demand, and potential fatalities across Australia. Importantly, this analysis shows that a 10% increase in the average SD level reduces the clinical burden approximately 3-fold, and the potential fatalities approximately 2-fold.

## 2. Methods

We utilised an agent-based model (ABM) for transmission and control of COVID-19 in Australia that has been developed in our previous work (1, 17) and implemented within a large-scale software simulator (AMTraC-19). The model was cross-validated with genomic surveillance data (6), and contributed to policy recommendations on social distancing that were broadly adopted by the World Health Organisation (18). The model separately simulates each individual as an agent within a surrogate population composed of about 23.4 million software agents. These agents are stochastically generated to match attributes of anonymous individuals (in terms of age, residence, gender, workplace, susceptibility, and immunity to diseases), informed by data from the Australian Census and the Australian Curriculum, Assessment and Reporting Authority. In addition, the simulation follows the known commuting patterns between the places of residence and work/study (19–21). Different contact rates specified within diverse social contexts (e.g., households, neighbourhoods, communities, and work/study environments) explicitly represent heterogeneous demographic and epidemic conditions (see Supplementary Material: Agent-based model). The model has previously been calibrated to produce characteristics of the COVID-19 pandemic corresponding to the ancestral lineage of SARS-CoV-2 (1, 17), using actual case data from the first and second waves in Australia, and re-calibrated for B.1.617.2 (Delta) variant using incidence data of the Sydney outbreak (see Supplementary Material: Model calibration).

Each epidemic scenario is simulated by updating agents' states in discrete time. In this work, we start from an initial distribution of infection, seeded by imported cases generated by the incoming international air traffic in Sydney's international airport (using data from the Australian Bureau of Infrastructure, Transport, and Regional Economics) (19, 20). At each time step during the seeding phase, this process probabilistically generates new infections within a 50 km radius of the airport (covering the area within Greater Sydney's boundaries), in proportion to the average daily number of incoming passengers (using a binomial distribution and data from the Australian Bureau of Infrastructure, Transport, and Regional Economics) (19).

A specific outbreak, originated in proximity to the airport, is traced over time by simulating the agents interactions within their social contexts, computed in 12-h cycles (“day” and “night.”) Once the outbreak size (cumulative incidence) exceeds a pre-defined threshold (e.g., 20 detected cases), the travel restrictions (TR) are imposed by the scenario, so that the rest of infections are driven by purely local transmissions, while no more overseas acquired cases are allowed (presumed to be in effective quarantine). Case-targeted non-pharmaceutical interventions (CTNPIs), such as case isolation (CI) and home quarantine (HQ), are applied from the outset. A scenario develops under some partial mass-vaccination coverage, implemented as either a progressive rollout, or a limited pre-pandemic coverage, as described in Supplementary Material: Vaccination modelling.

The outbreak-growth phase can then be interrupted by another, “suppression,” threshold (e.g., 100 or 400 cumulative detected cases) which triggers a set of general NPIs, such as social distancing (SD) and school closures (SC). Every intervention is specified *via* a macro-distancing level of compliance (i.e., *SD* = 0.8 means 80% of agents are socially distancing), and a set of micro-distancing parameters (quantifying context-specific interaction strengths, e.g., moderate or tight restrictions) that indicate the level of social distancing within a specific social context (households, communities, workplaces, etc.). For instance, for those agents that are compliant, contacts (and thus likelihood of infection) can be reduced during a lockdown to *SD*_*w*_ = 0.1 within workplaces and *SD*_*c*_ = 0.25 within communities, whilst maintaining contacts *SD*_*h*_ = 1.0 within households. To re-iterate, “social distancing” modelled in this study comprises a range of restrictions that reduce the intensity of interactions among individuals, including mask wearing, physical distancing by several metres, mobility, and so on. We do not estimate a relative importance of these specific NPI approaches, each of which separately contributes to reducing SARS-CoV-2 transmission (22–27), focusing instead on a differentiation between the effects of NPIs and vaccination campaigns.

## 3. Results

**Figure 1:**
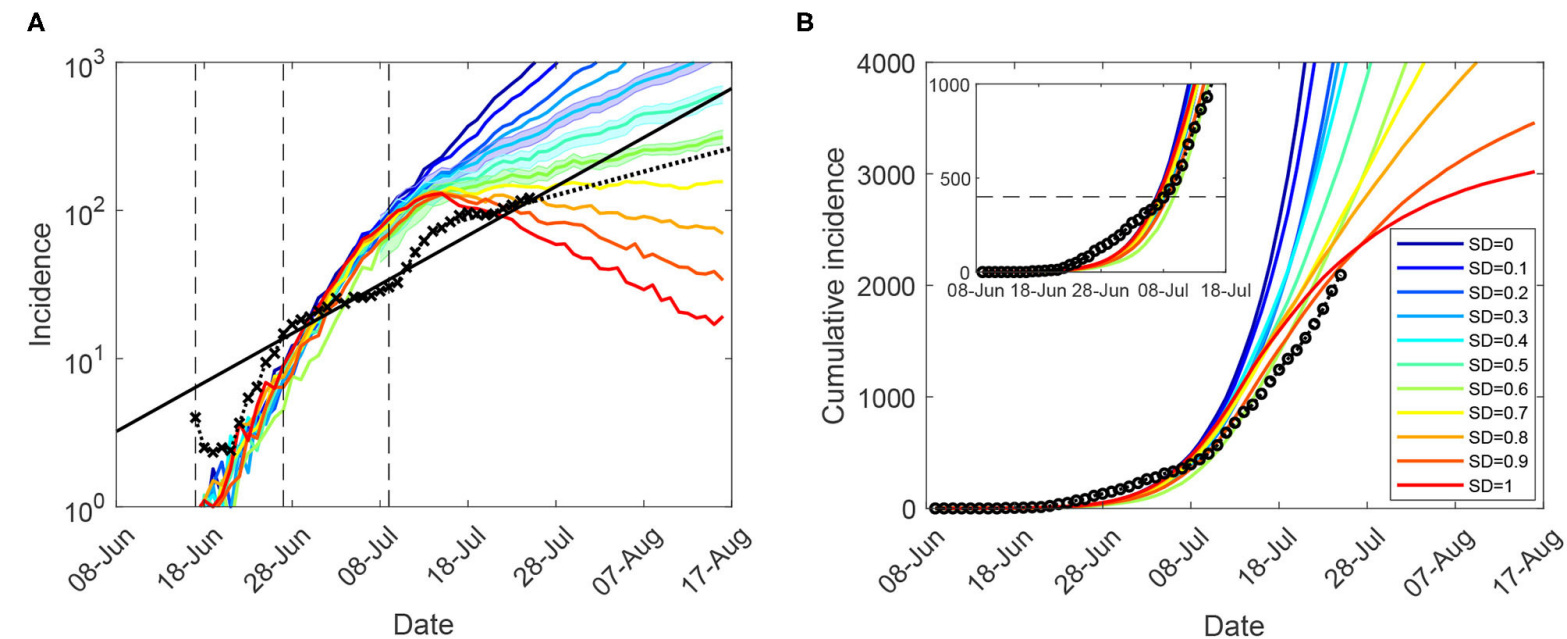
Moderate restrictions (NSW; progressive vaccination rollout; suppression threshold: 400 cases): a comparison between simulation scenarios and actual epidemic curves, under moderate interaction strengths (*CI*_*c*_ = *CI*_*w*_ = 0.25, *HQ*_*c*_ = *HQ*_*w*_ = 0.25, *SD*_*c*_ = 0.25, *SC* = 0.5). A moving average of the actual time series up to 25 July for **(A)** (log-scale) incidence (crosses), and **(B)** cumulative incidence (circles); with an exponential fit of the incidence's moving average (black solid: β_*II*_, and black dotted: β_*III*_). Vertical dashed marks align the simulated days with the outbreak start (17 June, day 9), initial restrictions (27 June, day 19), and tighter lockdown (9 July, day 31). Traces corresponding to each social distancing (SD) compliance level are shown as average over 10 runs (coloured profiles for SD varying in increments of 10%, i.e., between *SD* = 0.0 and *SD* = 1.0). 95% confidence intervals for incidence profiles, for *SD*∈{0.4, 0.5, 0.6}, are shown as shaded areas. Each SD intervention, coupled with school closures, begins with the start of tighter lockdown, when cumulative incidence exceeds 400 cases (**B**: inset). The alignment between simulated days and actual dates may slightly differ across separate runs. Case isolation and home quarantine are in place from the outset.

Using the ABM calibrated to the Delta (B.1.617.2) variant, we varied the macro- and micro-parameters (for CI, HQ, SC, and SD), aiming to match the incidence data recorded during the Sydney outbreak in a *retrodiction* mode. As shown in Figure 1, the modelling horizon was set to July 25 and assumed a progressive vaccination rollout in addition to a tighter lockdown being imposed at 400 cases (corresponding to July 9). Construction works were temporarily paused across Greater Sydney during 19–30 July 2021 (inclusive), with the temporary “construction ban” lifted on 28 July (28, 29). Within the considered timeline, the actual incidence growth rate has reduced from β_*I*_ = 0.098 (17 June – 13 July), to β_*II*_ = 0.076 (17 June – 25 July), to β_*III*_ = 0.037 (16–25 July), as detailed in Supplementary Material: Growth rates.

The closest match to the actual incidence data over the entire period was produced by a moderate macro-level of social distancing compliance, *SD* = 0.5, or even a lower level (*SD* = 0.4) for the period up to 13 July (see Figure 1 and Supplementary Material: Sensitivity of outcomes for moderate restrictions, Supplementary Figure 2; also see section 4 for a comparison of these SD levels with real-world mobility reductions). The match is not exact—with the actual incidence growth rate changing several times during this period—perhaps as a consequence of restrictions being imposed heterogeneously across different local government areas. Importantly, however, the growth in actual incidence during the period of the comprehensive lockdown restrictions (16–25 July) is best matched by a higher compliance level, *SD* = 0.6. This match is also reflected by proximity of the corresponding growth rate β_0.6_ = 0.029 to the incidence growth rate β_*III*_ = 0.037. The considered SD levels were based on moderately reduced interaction strengths within community, i.e., *SD*_*c*_ = 0.25, see Table 1, which were inadequate for outbreak suppression even with high macro-distancing such as *SD* = 0.7.

**Table 1:** The macro-distancing parameters and interaction strengths: retrodiction (“moderate”) and counter-factual (“tight.”).

	**Macro-distancing**	**Interaction strengths**		
**Intervention**	**Compliance levels**	**Household**	**Community**	**Workplace/School**
	**moderate → high**		**moderate → tight**	**moderate → tight**
CI	0.7	1.0	0.25 → 0.1	0.25 → 0.1
HQ	0.5	2.0	0.25 → 0.1	0.25 → 0.1
SC (children)	1.0	1.0	0.5 → 0.1	0
SC (parents)	0.5	1.0	0.5 → 0.1	0
SD	0.4 → 0.8	1.0	0.25 → 0.1	0.1

**Figure 2:**
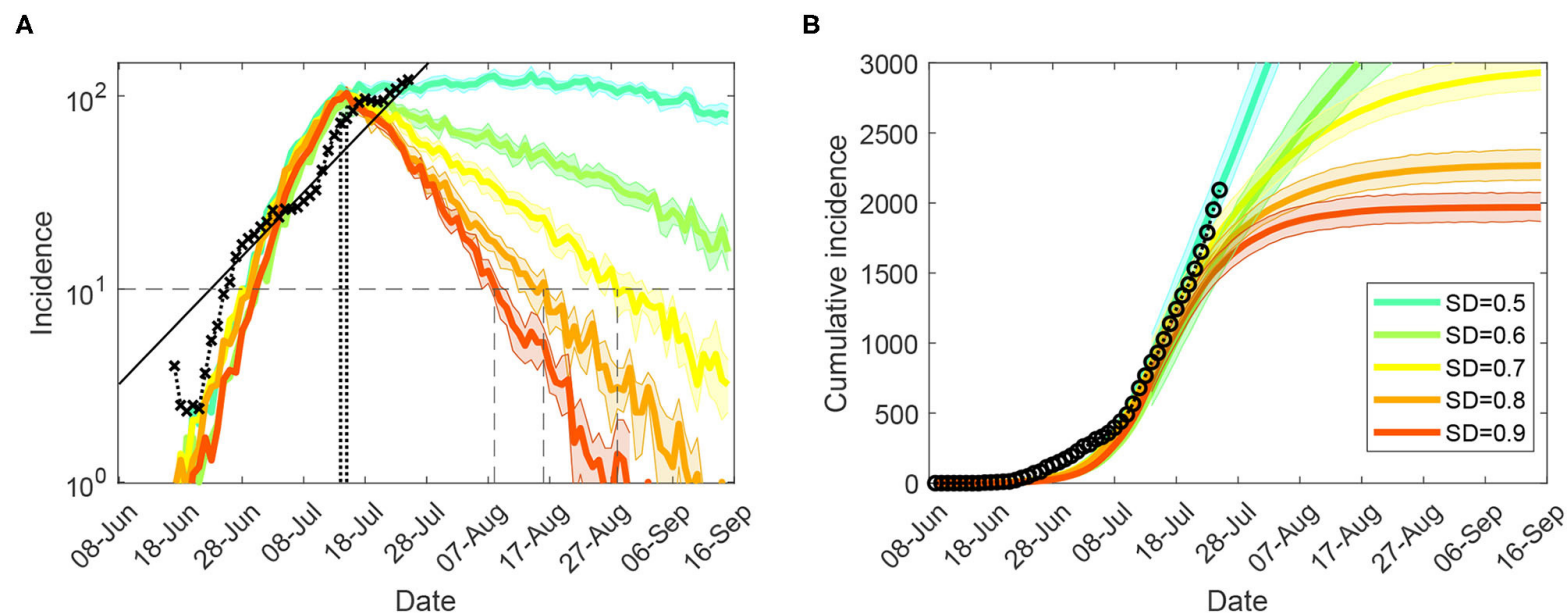
Tight restrictions (NSW; progressive vaccination rollout; suppression threshold: 400 cases): counter-factual simulation scenarios, under lowest feasible interaction strengths (*CI*_*c*_ = *CI*_*w*_ = 0.1, *HQ*_*c*_ = *HQ*_*w*_ = 0.1, *SD*_*c*_ = 0.1, *SC* = 0.1), for **(A)** (log scale) incidence (crosses), and **(B)** cumulative incidence (circles). Traces corresponding to feasible social distancing (SD) compliance levels are shown as average over 10 runs (coloured profiles for SD varying in increments of 10%, i.e., between *SD* = 0.5 and *SD* = 0.9). Vertical lines mark the incidence peaks (dotted) and reductions below 10 daily cases (dashed). Each SD intervention, coupled with school closures, begins with the start of tighter lockdown, when cumulative incidence exceeds 400 cases (i.e., simulated day 31). The alignment between simulated days and actual dates may slightly differ across separate runs. Case isolation and home quarantine are in place from the outset.

Furthermore, we considered moderate-to-high macro-levels of social distancing, 0.5 ≤ *SD* ≤ 0.9, while maintaining *CI* = 0.7 and *HQ* = 0.5, in a *counter-factual* mode by reducing the micro-parameters (the interaction strengths for CI, HQ, SC, and SD) within their feasible bounds. Again, the control measures were triggered by cumulative incidence exceeding 400 cases (corresponding to a tighter lockdown imposed on July 9). An effective suppression of the outbreak within a reasonable timeframe is demonstrated for macro-distancing at *SD*≥0.7, coupled with the lowest feasible interaction strengths for most interventions, i.e., *NPI*_*c*_ = 0.1 (where NPI is one of CI, HQ, SC, and SD), as shown in Figure 2 and summarised in Table 1. For *SD* = 0.8, new cases fall below 10 per day approximately a month (33 days) after the peak in incidence, while for *SD* = 0.7 this period reaches 45 days^2^. Social distancing at *SD* = 0.9 is probably infeasible (as this assumes that 90% of the population consistently stays at home), but would reduce the new cases to below 10 a day within four weeks (25 days) following the peak in incidence.

Supplementary Material (Sensitivity of suppression outcomes for tight restrictions) presents results obtained for the scenarios which assume a limited pre-pandemic vaccination coverage (immunising 6% of the population). A positive impact of the partial progressive rollout which covers up to 40% of the population by mid-September is counterbalanced by a delayed start of the tighter lockdown, with the 12-day delay leading to a higher peak-incidence, as can be seen by comparing Figure 2 and Supplementary Figure 4. For example, for *SD* = 0.8, a scenario following the limited pre-pandemic vaccination, but imposing control measures earlier, demonstrates a reduction of incidence below 10 daily cases in four weeks after the peak in incidence (Supplementary Figure 4), rather than 33 days under progressive rollout (Figure 2). For *SD* = 0.9 the suppression periods differ by about one week: 17 days (Supplementary Figure 4) against 25 days (Figure 2). However, this balance is nonlinear, as shown in Table 2: for *SD* = 0.7, the suppression period under the pre-pandemic vaccination scenario approaches 55 days (Supplementary Figure 4), in contrast to the progressive rollout scenario achieving suppression earlier, in 45 days (Figure 2). This is, of course, explained by the longer suppression period under *SD* = 0.7, during which a progressive rollout makes a stronger impact.

**Table 2:** Comparison of control measures: projected lockdown duration after the incidence peak, until new cases fall below 10 per day.

**Vaccination**	**Vaccination**	**Lockdown trigger**	**Post-peak duration (days)**		
**scenario**	**uptake**	**(cumulative cases)**	***SD* = 0.7**	***SD* = 0.8**	***SD* = 0.9**
Pre-pandemic	6%	100	55	28	17
Progressive	→ 40%	400	45	33	25

**Figure 3:**
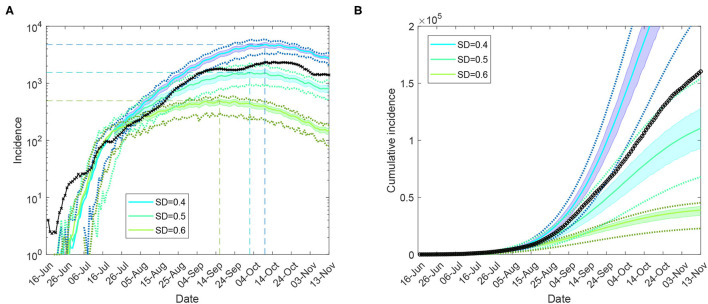
Moderate restrictions (Australia; progressive vaccination rollout; suppression threshold: 400 cases): a comparison between simulation scenarios and actual epidemic curves up to November 13, under moderate interaction strengths (*CI*_*c*_ = *CI*_*w*_ = 0.25, *HQ*_*c*_ = *HQ*_*w*_ = 0.25, *SD*_*c*_ = 0.25, *SC* = 0.5). A moving average of the actual time series for **(A)** (log scale) incidence (crosses), and **(B)** cumulative incidence (circles). Traces corresponding to social distancing levels *SD*∈{0.4, 0.5, 0.6} are shown for the period between 16 June and 13 November, as averages over 10 runs (colored profiles). 95% confidence intervals are shown as shaded areas. For each SD level, minimal and maximal traces, per time point, are shown with dotted lines. Peaks formed during the suppression period for each SD profile are identified with coloured dashed lines. Each SD intervention, coupled with school closures, begins with the start of initial restrictions. The alignment between simulated days and actual dates may slightly differ across separate runs. Case isolation and home quarantine are in place from the outset.

We then considered feasible scenarios tracing the epidemic spread at the national level for the period between mid-June and mid-November 2021, constrained by moderate levels of social distancing, *SD*∈{0.4, 0.5, 0.6}, under partial CTNPIs (*CI* = 0.7 and *HQ* = 0.5), see Supplementary Table 4. A progressive vaccination rollout was simulated concurrently with the continuing restrictions (see Supplementary Material: Vaccination modelling). Our Australia-wide model was calibrated by 31 August 2021, adopting a higher fraction of symptomatic children, σ_*c*_ = 0.268 (see Supplementary Material: Model calibration). The actual incidence curve is traced between the profiles formed by *SD* = 0.4 and *SD* = 0.5, with the latter providing the best match. The model projection for incidence peaking across the nation in the range between approximately 1,500 and 5,000 daily cases pointed to early to mid-October. This projection is validated by the actual profiles, as shown in Figure 3 and Supplementary Figure 11. The corresponding levels of simulated and actual vaccination coverage reached across Australia are shown in Supplementary Material: Vaccination modelling.

Using the Australia-wide model, we quantified the expected demand in terms of hospitalisations (occupancy) and the intensive care units (ICUs), and the number of potential fatalities across the nation. The estimation methods are described in Supplementary Material: Hospitalisations and fatalities. The projections obtained for the three feasible levels of social distancing, *SD*∈{0.4, 0.5, 0.6}, are shown in Supplementary Figures 8–10, and summarised in Table 3 and Supplementary Tables 9, 10. The scenario developing under *SD* = 0.5 offers the best match with the actual dynamics again. As expected, the unvaccinated cases form a vast majority among the hospitalisations, ICU occupancy and fatalities (cf. Supplementary Tables 9, 10). Importantly, a comparison across the three moderate levels of social distancing, *SD*∈{0.4, 0.5, 0.6} shows that with a 10% increase in the level of social distancing, the hospitalisations and ICU demand reduce approximately 3-fold, and the fatalities reduce at least two times. These effects of a 10% increase in the social distancing adherence on the clinical burden and the potential fatalities are robust with respect to changes in the vaccine efficacy against infectiousness, as shown in Supplementary Figure 12, and Tables 9, 10.

**Table 3:** Estimates (across Australia) of the peak demand in hospitalisations and ICUs; and cumulative fatalities (15 October 2021).

**Scenario**	**Peak hospitalisations:**	**Peak ICU demand:**	**Cumulative fatalities:**
	**mean and 95% CI**	**mean and 95% CI**	**mean and 95% CI**
*SD* = 0.4	4805 [4282, 5257]	812 [731, 885]	1201 [1057, 1326]
*SD* = 0.5	1604 [1358, 1844]	272 [230, 312]	539 [479, 624]
*SD* = 0.6	533 [476, 579]	91 [80, 99]	235 [209, 256]
Actual	1551 (28 September)	308 (12 October)	596 (15 October)

## 4. Discussion

Despite a relatively high computational cost, and the need to calibrate numerous internal parameters, ABMs capture the natural history of infectious diseases in a good agreement with the established estimates of incubation periods, serial/generation intervals, and other key epidemiological variables. Various ABMs have been successfully used for simulating actual and counter-factual epidemic scenarios based on different initial conditions and intervention policies (30–34).

Our early COVID-19 study (1) modelled transmission of the ancestral lineage of SARS-CoV-2 characterised by the basic reproduction number of *R*_0_≈3.0 (adjusted *R*_0_≈2.75). This study compared several NPIs and identified the minimal SD levels required to control the first wave in Australia. Specifically, a compliance at the 90% level, i.e., *SD* = 0.9 (with *SD*_*w*_ = 0 and *SD*_*c*_ = 0.5) was shown to control the disease within 13-14 weeks. This relatively high SD compliance was required in addition to other restrictions (TR, CI, HQ), set at moderate levels of both macro-distancing (*CI* = 0.7 and *HQ* = 0.5), and interaction strengths: *CI*_*w*_ = *HQ*_*w*_ = *CI*_*c*_ = *HQ*_*c*_ = 0.25, *CI*_*h*_ = 1.0, and *HQ*_*h*_ = 2.0 (1).

The follow-up work (17) quantified possible effects of a mass-vaccination campaign in Australia, by varying the extents of *pre-pandemic* vaccination coverage with different vaccine efficacy combinations. This analysis considered hybrid vaccination scenarios using two vaccines adopted in Australia: BNT162b2 (Pfizer/BioNTech) and ChAdOx1 nCoV-19 (Oxford/AstraZeneca). Herd immunity was shown to be out of reach even when a large proportion (82%) of the Australian population is vaccinated under the hybrid approach, necessitating future partial NPIs for up to 40% of the population. The model was also calibrated to the basic reproduction number of the ancestral lineage (*R*_0_≈3.0, adjusted *R*_0_≈2.75), and used the same moderate interaction strengths as the initial study (1) (except *SD*_*c*_ = 0.25, reduced to match the second wave in Melbourne in 2020).

In this work, we re-calibrated the ABM to incidence data from the ongoing third pandemic wave in Australia driven by the Delta variant. The reproductive number was calibrated to be at least twice as high (*R*_0_ = 5.97) as the one previously estimated for pandemic waves in Australia. We then explored effects of available NPIs on the outbreak suppression, under a *progressive vaccination* scenario. The retrodictive modelling identified that the current epidemic curves, which continued to grow (until mid-October 2021), can be closely matched by moderate social distancing coupled with moderate interaction strengths within community (*SD* in [0.4, 0.5], *SD*_*c*_ = 0.25), as well as moderate compliance with case isolation (*CI* = 0.7, *CI*_*w*_ = *CI*_*c*_ = 0.25) and home quarantine (*HQ* = 0.5, *HQ*_*w*_ = *HQ*_*c*_ = 0.25). The estimate of compliance has briefly improved to *SD*≈0.6 during the period of comprehensive lockdown measures, announced on July 17, but returned to *SD*≈0.5 in early August.

We note that the workers delivering essential services are exempt from lockdown restrictions. The fraction of the exempt population can be inferred conservatively as 4% (strictly essential) (35), more comprehensively as approximately 19% (including health care and social assistance; public administration and safety; accommodation and food services; transport, postal and warehousing; electricity, gas, water and waste services; financial and insurance services), but can reach more significant levels, around 33%, if all construction, manufacturing, and trade (retail/wholesale) are included in addition (36). The latter, broad-range, case limits feasible social distancing levels to approximately *SD*≈0.7. However, even with these inclusions, there is a discrepancy between the level estimated by ABM (*SD* in [0.4, 0.5]) and the broad-range feasible level (*SD*≈0.7). This discrepancy would imply that approximately 20–25% of the population have not been consistently complying with the imposed restrictions, while 30–35% may have been engaged in services deemed broadly essential (other splits comprising 50–60% of the “non-distancing” population are possible as well).

The inferred levels of social distancing are supported by real-world mobility data (37). Specifically, when compared to baseline (i.e., the median value for the corresponding day of the week, during the 5-week period 3 January–6 February 2020, as set by data provider to represent the pre-pandemic levels), the reports for July 16 showed 31% reduction of mobility at workplaces, and 37% reduction of mobility in retail and recreation settings, with concurrent 65% reduction of mobility on public transport. On July 21, the mobility reductions were reported as 43% (workplaces), 41% (retail and recreation), and 72% (public transport). The extent of the mobility reduction in workplaces, as well as retail and recreation, closely matched the social distancing levels estimated by the model (approximately 40%). The partial reductions in mobility across workplaces, retail, and recreation have since been maintained around 40–50% on average (37). According to numerous reports (38–40), the infection spread among essential workers was substantial, and the interactions within workplaces and community contributed to the disease transmission stronger than contacts in public transport.

Moderate levels of compliance (*SD* in [0.4, 0.6]) would be inadequate for suppression of even less transmissible coronavirus variants (1). The Delta variant demands a stronger compliance and a reduction in the scope of essential services (especially, in a setting with low immunity). Specifically, our results indicate that an effective suppression within a reasonable timeframe can be demonstrated only for very high compliance with social distancing (*SD*≥0.7), supported by dramatically reduced, and practically infeasible, interaction strengths within the community and work/study environments (*NPI*_*c*_ = *NPI*_*w*_ = 0.1). Importantly, a significant fraction of local transmissions during the Sydney outbreak in NSW, as well as during the following outbreak in Melbourne, VIC [which started on 13 July 2021, was initially suppressed, but then resumed its growth on 4 August 2021 (5)], occurred in the suburbs characterised by socioeconomic disadvantage profiles, as defined by The Australian Bureau of Statistics' Index of Relative Socio-economic Advantage and Disadvantage (IRSAD) (38, 39, 41). To a large extent, the epidemic spread in these suburbs was driven by structural factors, such as higher concentrations of essential workers, high-density housing, shared and multi-generational households, etc. Thus, even a combination of government actions (e.g., a temporary inclusion of some services previously deemed essential under the lockdown restrictions (28, 29), while providing appropriate financial support to the affected businesses and employees), and a moderate community engagement with the suppression effort, proved to be insufficient for the outbreaks' suppression.

Obviously, the challenges of suppressing emerging variants of concern can be alleviated by a growing vaccination uptake. However, in Australia, the vaccination rollout was initially limited by various supply and logistics constraints. Furthermore, as our results demonstrate, a progressive vaccination rollout reaching up to 40% of the population (i.e., approximately 50% of adults) was counter-balanced by a delayed introduction of the tighter control measures. This balance indicated that a comprehensive mass-vaccination rollout plays a crucial role over a longer term and should preferably be carried out in a pre-outbreak phase (17). Ultimately, the epidemic peak in NSW during the lockdown period was reached only when about a half of the adults were double vaccinated by mid-September (i.e., 49.6% on 15 September 2021) (16). Across the nation, the peak in incidence was observed by mid-October (as predicted by the model), once approximately two thirds of adults were double vaccinated (16), also in concordance with the model (see Supplementary Material: Vaccination modelling).

A post-lockdown increase in infections is expected when the stay-at-home orders are lifted in recognition of immunising 70%, and then 80%, of adults (42). However, a detailed analysis of a possible post-lockdown surge in infections, the resultant increased demand on the healthcare system, and potential fatalities, is outside of the scope for this study.

While the model was not directly used to inform policy, it forms part of the information set available to health departments, and we hope that its policy relevance can contribute to rapid and comprehensive responses in jurisdictions within Australia and overseas. A failure in reducing the size of the initial outbreak, due to a delayed vaccination rollout, challenging socioeconomic profiles of the primarily affected areas, inadequate population compliance, and a desire to maintain and restart socioeconomic activities, has generated a substantial pandemic wave affecting the entire nation (43–45).

### 4.1. Study Limitations

In modelling the progressive vaccination rollout, we assumed a constant weekly uptake rate of 3%, while the rollout was accelerating. The rate of progressive vaccination is expected to vary, being influenced by numerous factors, such as access to national stockpiles, dynamics of social behaviour, and changing medical advice. In addition, we did not consider a diminishing vaccine efficacy, given that the temporal scope of the study was limited to a relatively short period of 6 months (June–November 2021) during which a progressive rollout was modelled. Thus, only a relatively small fraction of the population vaccinated during the very first few months would be experiencing a tangibly diminished vaccine efficacy (with respect to the Delta variant) (46). Nevertheless, the study included a sensitivity analysis of the vaccine efficacy across three static levels.

Another limitation is that the surrogate ABM population which corresponds to the latest available Australian Census data from 2016 (23.4M individuals, with 4.45M in Sydney) is smaller than the current Australian population (25.8M, with 4.99M in Sydney). We expect low sensitivity of our results to this discrepancy due to the outbreak size being three orders of magnitude smaller than Sydney population.

Finally, the model does not directly represent in-hotel quarantine and in-hospital transmissions. Since the frontline professionals (health care and quarantine workers) were vaccinated in a priority phase carried out in Australia in early 2021, i.e., before the Sydney outbreak, this limitation is expected to have a minor effect. Overall, as the epidemiology of the Delta variant continues to be refined with more data becoming available, our results may benefit from a retrospective analysis.

## Data Availability Statement

We used anonymised data from the 2016 Australian Census obtained from the Australian Bureau of Statistics (ABS). These datasets can be obtained publicly, with the exception of the work travel data which can be obtained from the ABS on request. It should be noted that some of the data needs to be processed using the TableBuilder: https://www.abs.gov.au/websitedbs/censushome.nsf/home/tablebuilder. The actual incidence data are available from the health departments across Australia (state, territories, and national), and at: https://www.covid19data.com.au/. Other source and supplementary data, including simulation output files, are available at Zenodo (47). The source code of AMTraC-19 is also available at Zenodo (48).

## Author Contributions

MP conceived and co-supervised the study and drafted the original article. SC and MP designed the computational experiments, re-calibrated the model, and estimated hospitalisations, ICU occupancy, and potential fatalities. CZ implemented simulations of progressive vaccination and social distancing policies. SC carried out the computational experiments, verified the underlying data, and prepared all figures. All authors had full access to all the data in the study, contributed to the editing of the article, read, and approved the final article.

## Funding

This work was partially supported by the Australian Research Council grants DP220101688 and DP200103005 (MP and SC). Additionally, CZ was supported in part by National Health and Medical Research Council project grant (APP1165876). AMTraC-19 is registered under The University of Sydney's invention disclosure CDIP Ref. 2020-018.

## Conflict of Interest

The authors declare that the research was conducted in the absence of any commercial or financial relationships that could be construed as a potential conflict of interest.

## Publisher's Note

All claims expressed in this article are solely those of the authors and do not necessarily represent those of their affiliated organizations, or those of the publisher, the editors and the reviewers. Any product that may be evaluated in this article, or claim that may be made by its manufacturer, is not guaranteed or endorsed by the publisher.

## Footnotes

^1^In describing a “wave” we follow the definition based on two key features: (i) an epidemic wave comprises upward and/or downward periods; and (ii) the increase during an upward period, as well as the decrease during a downward period, must be substantial over a period of time (4).

^2^A post-peak period duration for each SD level is obtained using the incidence trajectory averaged over ten simulation runs.
