# Supplementary material for: Simulating Transmission Scenarios of the Delta Variant of SARS-CoV-2 in Australia
Source: Front Public Health. 2022 Feb 24;10:823043. doi: 10.3389/fpubh.2022.823043 (PMC8907620; doi:10.3389/fpubh.2022.823043)
Supplement: Supplementary file 1 [file Data_Sheet_1.pdf]

Supplementary Material

1 AGENT-BASED MODEL OF TRANSMISSION AND CONTROL OF THE COVID-19 PANDEMIC IN AUSTRALIA: AMTRAC-19

1.1 Demographics

Each agent in the artificial population belongs to several mixing groups stochastically generated from census data based on Statistical Areas (SA1 and SA2) level statistics, and the distributions across age groups, households and workplaces (Cliff et al., 2018; Zachreson et al., 2018; Fair et al., 2019; Chang et al., 2020). Agents are split into five different age groups: preschool aged children (0-4), children (5-18), young adults (19-29), adults (30-64) and older adults (65+), with a further refinement into specific ages derived when necessary from the census distribution. During the daytime simulation cycle (time-step), agents interact in “work regions”, e.g., an agent representing an adult individual (19-64) interacts within a work group, while children agents (5-18) interact within classrooms, grades, and schools. During the nighttime cycle, individuals interact in “home regions”, e.g., households, household clusters, neighbourhoods (SA1), and communities (SA2). Preschool children and older adults interact only in home regions during the nighttime simulation cycle. During weekends, the nighttime simulation cycle runs twice, thus replacing daytime interactions in work regions with an additional interaction cycle in home regions.

1.2 Transmission probability

At each time-step n the simulator computes the probability of infection $p_i(n)$ for a susceptible agent i. This is determined by considering all relevant mixing contexts (daytime or nighttime) g for the agent i, selected from $G_i(n)$, and the infection states of other agents j in each context A_g. The context-dependent probability that infectious individual j infects susceptible individual i in context g in a single time step, $p^g_{j\to i}$, is defined as follows:

$$p^g_{j\to i}(n) = \kappa \, f(n - n_j \mid j) \, q^g_{j\to i} \tag{S1}$$

where κ is a global scaling factor (selected to calibrate to the reproductive number R_0), n_j denotes the time when agent j becomes infected, and $q^g_{j\to i}$ is the probability of transmission from agent j to i at the infectivity peak, derived from the transmission or contact rates. The function $f : \mathbb{N} \to [0, 1]$ quantifies the infectivity of agent j over time, according the natural history of the disease; $f(n - n_j \mid j) = 0$ when $n < n_j$; cf. Supplementary Fig. S1. The transmission rates $q^g_{j\to i}$ for the household and study environments are shown in Table S1. The contact rates $c^g_{j\to i}$ for household clusters, neighbourhoods, and communities are detailed in Table S2. These contact rates are rescaled, using a fixed scaling factor ρ, to transmission rates (Cliff et al., 2018):

$$q^g_{j\to i} = \rho \, c^g_{j\to i}. \tag{S2}$$

The overall probability that a susceptible agent i is infected at a given time step n is then calculated as (Chang et al., 2020)

$$p_i(n) = 1 - \prod_{g\in G_i(n)} \left[\prod_{j\in A_g\setminus i} (1 - p^g_{j\to i}(n)) \right]. \tag{S3}$$

This expression is adjusted to account for the agents adhering to various non-pharmaceutical interventions and vaccinations, as detailed below, see (S4) and (S5). At the end of each cycle, a Bernoulli trial with probability $p_i(n)$ determines if a susceptible agent becomes infected.

1.3 Natural history of disease

In a single agent, the disease progression from exposure to recovery develops over several agent states: SUSCEPTIBLE, LATENT, infectious SYMPTOMATIC, infectious ASYMPTOMATIC, and RECOVERED. In general, the first phase is the latent period during which infected agents are unable to infect others. However, in modelling the Delta variant, AMTraC-19 sets this period to zero days. The second phase is the incubation period characterised by an exponentially increasing infectivity, from 0% to 100%, reaching its peak at the end of the incubation period after T_{inc} days (see Supplementary Fig. S1). In the third, post-incubation, phase the infectivity decreases linearly from the peak to zero, over T_{rec} days until the recovery (with immunity). The parameters $T_{inc}(i)$ and $T_{rec}(i)$ are randomly generated for each agent i, see Table S3, thus defining the disease progression in the affected agent, i.e., $D(i) = T_{inc}(i) + T_{rec}(i)$.

Asymptomatic cases are set to be 50% as infectious as symptomatic cases, $\alpha = 0.5$. We assume that 67% of adult cases are symptomatic ($\sigma_a = 0.67$), and a lower fraction (either 13.4% or 26.8%) is set as symptomatic in children (e.g., $\sigma_c = 0.268$). The fractions $\sigma_{a,c}$ reduce the probability of becoming ill (symptomatic) $p_i^d(n)$, given the infection probability $p_i(n)$, for each adult or child agent: $p_i^d(n) = \sigma_{a|c} p_i(n)$. On each simulated day, pre-symptomatic, symptomatic and asymptomatic cases are detected with specific probabilities r (see Supplementary Material: Model calibration). Only detected cases are counted in the incidence profiles. Table S3 summarises the main model parameters.

1.4 Reproductive number

For every scalar κ, the reproductive number R_0 is estimated numerically (Zachreson et al., 2020), by stochastic sampling of index cases (sample size $\approx 10^4$). Every micro-simulation randomly selects a single index case, and detects the number of secondary infections generated during the period until the index case is recovered. The secondary cases themselves are prevented from generating further infections, so that all detected cases are attributed to the index case. In order to reduce the bias in selecting a typical (rather than purely random) index case, we employ "the attack rate pattern weighted index case" method (Germann et al., 2006; Zachreson et al., 2020), based on age-specific attack rates. These age-stratified weights, computed as averages over many full simulation runs, are assigned to secondary cases produced by the micro-simulation sample of index cases. This accounts for the correlations between age groups and population structure (Miller, 2009). Given the five age groups, [0–4, 5–18, 19–29, 30–64, 65+], the following age-specific weights were used in producing R_0 as the weighted average of secondary cases: [0.064, 0.1919, 0.1412, 0.4583, 0.1446].

This procedure is different from an empirical estimation of the reproductive number R_0 which would require comprehensive and still unavailable data on the actual secondary infections generated by different index cases. Instead, the numerical estimations of R_0 derived from the ABM are compared with the known estimates of R_0, and further validated by checking the concordance between projected and actual epidemic curves (see Supplementary Material: Model calibration).

1.5 Non-pharmaceutical interventions

The agents affected by various NPIs (case isolation: CI; home quarantine: HQ; school closures: SC; social distancing: SD) are determined in the beginning of each simulation run, given specific compliance levels explored by a simulation scenario. Every intervention F is specified via the fraction F of the population complying with the NPI ("macro-distancing"), and a set of interaction strengths F_g ("micro-distancing") that modify the transmission probabilities within a specific mixing context g: households (F_h), communities (F_c), and workplaces/study environments (F_w). For non-complying agents j, the interaction strengths are unchanged, i.e., $F_g(j) = 1$, while for complying agents j, the strengths are generally different: $F_g(j) \neq 1$, so that the transmission probability of infecting a susceptible agent i is adjusted as follows:

$$p_i(n) = 1 - \prod_{g \in G_i(n)} \left[\prod_{j \in A_g \setminus i} \left(1 - F_g(j)\, p^g_{j \to i}(n)\right) \right]. \tag{S4}$$

The intervention-induced restrictions are applied in a specific order: CI, HQ, SD, SC (for parents SC^a and children SC^c), with only the most relevant interaction strength F_g applied during each simulation cycle. For example, if a symptomatic student is in case isolation, then the interaction strengths HQ_g, SD_g and SC^c_g would not modify the agent's transmission probabilities, even if this agent is considered compliant with the corresponding measures, and the only applicable strength would be CI_g. The macro-distancing levels of compliance and the interaction strengths (micro-parameters) defining the NPIs are summarised in Table 1. To re-iterate, the macro-distancing compliance levels across interventions F define how many agents (i.e., F) adjust their interaction strengths to the micro-distancing levels F_g within specific contexts g.

At macro-level, some interventions, e.g., the CI and HQ measures, are set to last during the full course of the simulated scenario. The duration of SD and/or SC measures varies. In general, there may be a predefined set of intervals describing the (possibly interrupted and resumed) duration of intervention F. In AMTraC-19, we express the continuous duration as the number of days, F_T, following a threshold F_X in cumulative detected cases. At micro-level, the interaction strengths are reduced during the same period F_t for most of the measures, except HQ which is modelled to reduce the interaction strengths of the compliant agents for 14 days. The micro-duration of CI is limited by the disease progression in the affected agent i, i.e., $D(i)$. Formally, an intervention F is defined by a set of parameters: $\{F, F_T, F_X, F_h, F_c, F_w, F_t\}$, for example, SD may be defined by $\{0.6, 191, 400, 1.0, 0.25, 0.1, 191\}$. A scenario is then defined by a combination of these sets defined for all interventions CI, HQ, SD, SC^a and SC^c, see Table S4.

1.6 Vaccination modelling

The national COVID-19 vaccine rollout strategy pursued by the Australian Government follows a hybrid approach combining two vaccines: BNT162b2 (Pfizer/BioNTech) and ChAdOx1 nCoV-19 (Oxford/AstraZeneca), administered across specific age groups (the eligibility policy has underwent multiple changes, with different age groups provided access progressively). Our model accounts for differences in vaccine efficacy for the two vaccines approved for distribution in Australia, and distinguishes between separate vaccine components: efficacy against susceptibility (VEs), disease (VEd) and infectiousness (VEi).

Given these components, the transmission probability of infecting a susceptible agent i is adapted as follows:

$$p_i(n) = (1 - \mathrm{VEs}_i)\left(1 - \prod_{g \in G_i(n)}\left[\prod_{j \in A_g \setminus i}(1 - (1 - \mathrm{VEi}_j)F_g(j)\, p^g_{j \to i}(n))\right]\right) \tag{S5}$$

where for vaccinated agents $\mathrm{VEi}_j = \mathrm{VEi}$ and $\mathrm{VEs}_i = \mathrm{VEs}$, and for unvaccinated agents $\mathrm{VEi}_j = \mathrm{VEs}_i = 0$. The probability of becoming ill (symptomatic) is affected by the efficacy against disease (VEd) as follows: $p^d_i(n) = (1 - \mathrm{VEd})\sigma_{a|c}p_i(n)$ for adults and children.

For the pre-pandemic vaccination rollout, the extent of pre-outbreak vaccination coverage was set at 6% of the population, approximately matching the level actually achieved in Australia by mid-June 2021. For the progressive vaccination rollout, the initial coverage was set at zero, followed by vaccination uptake averaging 3% per week for the duration of simulation, and reaching the levels detailed in Table S5. Specifically, a policy-relevant milestone of 70% vaccinated adults is reached within the model around 13 November, that is, after 121 days of social distancing and school closures, see Supplementary Data 2; cf. Table S4 for duration of measures.

In setting the efficacy of vaccines against B.1.617.2 (Delta) variant, we followed the study of Bernal et al. (Bernal et al., 2021) which estimated the efficacy of BNT162b2 (Pfizer/BioNTech) as VEc $\approx$ 0.9 (more precisely, 87.9% with 95% CI: 78.2 to 93.2), and the efficacy of ChAdOx1 nCoV-19 (Oxford/AstraZeneca) as VEc $\approx$ 0.6 (i.e., 59.8% with 95% CI: 28.9 to 77.3). Given the constraint for the clinical efficacy (Zachreson et al., 2021):

$$\mathrm{VEc} = \mathrm{VEd} + \mathrm{VEs} - \mathrm{VEs}\ \mathrm{VEd}, \tag{S6}$$

we set VEd= VEs= 0.684 for BNT162b2, and VEd= VEs= 0.368 for ChAdOx1 nCoV-19.

Recent studies also provided the estimates of efficacy against infectiousness (VEi) for both considered vaccines at a level around 0.5 (Harris et al., 2021). A general sensitivity analysis of the model to changes in VEi and VEc was carried out in (Zachreson et al., 2021).

In both rollout scenarios, the vaccinations are assumed to be equally balanced between the two vaccines, so that each type is given to approximately (i) 0.7M individuals initially, by mid-June, or (ii) 4.7M individuals progressively, by mid-September. Vaccines are distributed according to specific age-dependent allocation ratios, $\approx$ 2547:30,000:1000, mapped to age groups [age ≥ 65] : [$18 \leq$ age < 65] : [age < 18], as explained in our prior work (Zachreson et al., 2021). That is, for every 2547 agents aged over 64 years, 30,000 individuals aged between 18 and 64 years, and 1000 agents under the age of 18 years are immunised. The allocation ratios are aligned with the age distribution of the Australian population (based on the 2016 ABS Census), while reflecting the tighter regulations on vaccine approval for children. At each simulation cycle this process immunises agents at a fixed rate. The allocations continue over a number of cycles until all adult agents are immunised. At the end of all allocations, the fraction of immunised children reaches approximately 20% of all children (i.e., agents under the age of 18 years).

2 MODEL CALIBRATION

In order to model transmission of the Delta (B.1.617.2) variant during the Sydney outbreak of COVID-19 (June–July 2021), we re-calibrated the model to match the reproduction number approximately twice as high as our previous estimates ($R_0 \approx 3.0$) for the two waves in Australia in 2020. In aiming at this level, we followed global estimates, which showed that the R_0 for B.1.617.2 is increased by 97% (95%

CI of 76–117%) relative to its ancestral lineage (Campbell et al., 2021). As implemented in our model, the re-calibrated reproductive number was estimated to be $R_0 = 5.97$ with a 95% CI of 5.93–6.00. The corresponding generation period is estimated to be $T_{gen} = 6.88$ days with a 95% CI of 6.81–6.94 days. The fraction of symptomatic children among all pediatric cases was set to $\sigma_c = 0.134$. The 95% confidence intervals (CIs) were constructed from the bias corrected bootstrap distribution (Efron and Tibshirani, 1994).

The model calibration varied the scaling factor κ (which scales age-dependent contact and transmission rates) in increments of 0.1. The best matching κ was identified when the resultant reproductive number, estimated in this work using age-stratified weights (Zachreson et al., 2020), was close to $R_0 = 6.0$. The procedure resulted in the following parametrisation:

- the scaling factor $\kappa = 5.3$ produced $R_0 = 5.97$ with 95% CI of 5.93–6.00 ($N = 6318$, randomly re-sampled in 100 groups of 100 samples; confidence intervals constructed by bootstrapping with the bias-corrected percentile method (Efron and Tibshirani, 1994));
- the fraction of symptomatic cases was set as $\sigma_a = 0.67$ for adults, and $1/5$ of that, i.e., $\sigma_c = 0.134$, for children;
- different transmission probabilities for asymptomatic/presymptomatic and symptomatic agents were set as “asymptomatic infectivity” (factor of 0.5) and “pre-symptomatic infectivity” (factor of 1.0) (Ferretti et al., 2020; Wu et al., 2020);
- incubation period T_{inc} was chosen to follow log-normally distributed incubation times with mean 4.4 days ($\mu = 1.396$ and $\sigma = 0.413$) (Zhang et al., 2021);
- a post-incubation infectious asymptomatic or symptomatic period was set to last between 7 and 14 days (uniformly distributed) (Centers for Disease Control and Prevention, 2020; Arons et al., 2020; Wölfel et al., 2020); and
- different detection probabilities were set as symptomatic (detection per day is 0.227) and asymptomatic/pre-symptomatic rates (detection per day is 0.01) (Zachreson et al., 2021).

Calibration of the fraction of symptomatic cases in children, including its higher setting $\sigma_c = 0.268$, is detailed in Supplementary Material: Sensitivity analysis. Estimation of the growth rates in incidence is described in Supplementary Material: Growth rates. In summary, two parameters were varied during the calibration: the scaling factor κ and the fraction of symptomatic cases in children σ_c, both reflecting specifics of the Delta variant. Other key epidemiological parameters listed above, e.g., different transmission probabilities, were calibrated previously (Chang et al., 2020; Zachreson et al., 2021). The remaining parameters describing the natural history of the disease, e.g., the incubation period, were set according to the available epidemiological evidence and varied during a sensitivity analysis, as described below. Table S6 summarises the key calibration outcomes.

The parametrisation of NPIs is independent of the disease model implemented in AMTraC-19, and thus, the NPI parameters were kept constant during calibration, being varied only within different scenarios (i.e., in setting moderate versus tight restrictions). As a result, the reported findings quantifying specific effects of interventions are decoupled from the disease model calibration.

3 SENSITIVITY ANALYSIS

Several internal parameters have been varied during prior sensitivity analyses (Chang et al., 2020; Zachreson et al., 2021). For this study, we carried out additional sensitivity analyses in terms of the incubation period, the reproductive number, the generation period, and the fraction of symptomatic cases for children σ_c. The

analysis presented below covers the time period between 17 June and 13 July inclusively, and is based on the pre-pandemic vaccination rollout. It can be contrasted with the progressive vaccination rollout studied in the main manuscript.

3.1 Incubation period

While previously the incubation period of COVID-19 was estimated to be distributed with the mean 5.5 days (Ferretti et al., 2020; Lauer et al., 2020), a more recent study of the Delta variant reported a shorter mean incubation period: 4.4 days (with 95% CI of 3.9-5.0) (Zhang et al., 2021). Our previous sensitivity analysis (Chang et al., 2020) showed that the model is robust to changes in the time to peak infectivity, investigated in the range between 4 and 7 days. Here we investigated the sensitivity of the updated model to changes in the incubation period specifically, varying it between the mean 4.4 days (log-normally distributed with $\mu = 1.396$ and $\sigma = 0.413$), matching the estimates of Zhang et al. (Zhang et al., 2021) and the mean 5.5 days (log-normally distributed with $\mu = 1.644$, $\sigma = 0.363$) (Ferretti et al., 2020).

The comparison between the 4.4-day and 5.5-day incubation periods was carried out for the same scaling factor $\kappa = 5.3$. The corresponding reproductive number changed from $R_0 = 5.97$ (95% CI of 5.93–6.00, $N = 6318$, $T_{inc} = 4.4$) to $R_0 = 6.39$ (95% CI of 6.36–6.43, $N = 7804$, $T_{inc} = 5.5$), that is, by approximately 7%. Similarly, the corresponding generation periods changed from $T_{gen} = 6.88$ (95% CI of 6.81–6.94, $N = 6318$, $T_{inc} = 4.4$) to $T_{gen} = 7.77$ (95% CI of 7.71–7.83, $N = 7804$, $T_{inc} = 5.5$), i.e., by approximately 13%. This relatively small sensitivity is explained by the high level of infectivity exhibited in our model by pre-symptomatic and asymptomatic individuals, see. Fig. S1.

3.2 Sensitivity of outcomes for moderate restrictions

Furthermore, using the suppression threshold of 100 cases, corresponding to the initial restrictions (June 27), we contrasted the scenarios based on different incubation periods. In doing so, we also varied global scalars κ producing different reproductive numbers and generation periods, thus extending the sensitivity analysis beyond local sensitivities. Specifically, for $T_{inc} = 5.5$, the scaling factor $\kappa = 5.0$ produced the reproductive number $R_0 = 6.09$ with 95% CI of 6.03–6.15 ($N = 6703$), and the generation period $T_{gen} = 7.74$ with 95% CI of 7.68–7.81. For each setting, we identified the levels of social distancing (SD), triggered by the suppression threshold of 100 cases (June 27), that best matched the actual incidence data. This comparison allowed us to establish robustness of the model outcomes to changes in T_{inc}, R_0 and T_{gen}. The outcomes are shown in Fig. S2 ($T_{inc} = 4.4$ and $R_0 = 5.97$, $T_{gen} = 6.88$, produced by $\kappa = 5.3$) and Fig. S3 ($T_{inc} = 5.5$ and $R_0 = 6.09$, $T_{gen} = 7.74$, produced by $\kappa = 5.0$).

The SD levels were based on moderately reduced interaction strengths detailed in Table 1. For the setting with shorter incubation period, the best matching scenarios were given by $SD = 0.4$ and $SD = 0.5$, see Fig. S2, with growth rate $\beta_{0.4} = 0.093$ being the closest match to the actual growth rate $\beta_I = 0.098$ (see Table S7). For the setting with longer incubation period, the best matching scenarios were produced by $SD = 0.3$ and $SD = 0.4$, see Fig. S3, with $\beta_{0.3} = 0.099$ being the closest match to β_I, while $\beta_{0.4} = 0.084$ was within the range. The sensitivity analysis revealed that the model outcomes for moderate restrictions are not strongly influenced by changes in T_{inc}, R_0 and T_{gen} within the explored ranges. In other words, it confirmed the conclusion that the social distancing compliance, at least until July 13, has been followed only moderately (around $SD = 0.4$), and would be inadequate to suppress the outbreak.

3.3 Sensitivity of suppression outcomes for tight restrictions (counter-factual analysis)

We also contrasted the suppression scenarios based on different incubation periods, reproductive numbers and generation periods (again using the threshold of 100 cases, triggered by the tight restrictions, under a pre-pandemic vaccination coverage). We explored feasible SD levels, $0.5 \leq SD \leq 0.9$, staying with $CI = 0.7$ and $HQ = 0.5$, but using the lowest feasible interaction strengths ($NPI_c = 0.1$, where NPI is one of CI, HQ, SC and SD), as specified in Table 1. For each setting, we identified the duration of measures required to reduce the incidence below 10. The results are shown in Fig. S4 ($T_{inc} = 4.4$, $R_0 = 5.97$, $T_{gen} = 6.88$, $\kappa = 5.3$) and Fig. S5 ($T_{inc} = 5.5$, $R_0 = 6.09$, $T_{gen} = 7.74$, $\kappa = 5.0$).

For each setting, a suppression of the outbreak is observed only for macro-distancing at $SD \geq 0.7$. Specifically, at $SD = 0.8$, new cases reduce below 10 per day approximately a month after a peak in incidence (when $T_{inc} = 5.5$, $R_0 = 6.09$), and the alternate setting ($T_{inc} = 4.4$, $R_0 = 5.97$) achieves this target a few days earlier (in 28 days). At $SD = 0.7$ the difference between the settings grows: while for the setting with $T_{inc} = 5.5$, $R_0 = 6.09$ the post-peak suppression period exceeds two months, the alternative ($T_{inc} = 4.4$, $R_0 = 5.97$) approaches the target about eight weeks (55 days) after the peak. There is a minor difference between the considered settings at $SD = 0.9$ which would achieve the required reduction within three weeks following the peak in incidence. The sensitivity analysis shows that changes in T_{inc}, R_0 and T_{gen} within the considered ranges do not strongly affect the modelled suppression outcomes. This supports the projection that the peak in incidence would be followed by approximately four weeks at $SD = 0.8$, and that this period would lengthen at least twice if the compliance reduced by 10% to $SD = 0.7$ (this setting produced the highest sensitivity among the levels demonstrating the suppression, due to its low rate of the incidence decline).

3.4 Fraction of symptomatic cases in children

During the initial outbreak in Sydney, several COVID-19 cases have been reported in schools and early childhood centres, with one outbreak in a primary school at the end of June involving four children (Danchin et al., 2021; Health Protection NSW, 2021a). On 8 September 2021, the National Centre for Immunisation Research and Surveillance (NCIRS) released a comprehensive report reviewing SARS-CoV-2 transmission within schools and early childhood services in NSW (NCIRS, 2021). The NCIRS report analysed transmissions of the Delta variant during the period between 16 June 2021 and 31 July 2021, with follow-up data until 19 August 2021. It noted that the majority of children (98%) had asymptomatic or mild infection, without further separating these two categories. Importantly, between 16 June and 31 July, 22% cases were recorded in children and young people under the age of 18 years (for the period between 1 and 19 August, this proportion was reported as 29%). The average fraction of cases in children, $A_c = 0.27$, within the range of [0.22, 0.29], is higher than the one reported in 2020 for the ancestral lineage of SARS-CoV-2 (with the corresponding proportion reported to be as low as 3.2%) (Macartney et al., 2020), suggesting a higher fraction of symptomatic cases for children (including mild cases).

We analysed the model sensitivity to changes in the fraction of symptomatic cases for children, varying it from $\sigma_c = 0.134$ to a higher value ($\sigma_c = 0.268$). This analysis was carried out for the incubation period $T_{inc} = 4.4$ days and the scalar $\kappa = 5.3$.

The reproductive number increased from $R_0 = 5.97$ for the lower fraction (95% CI of 5.93–6.00, over $N = 6318$ simulations), to $R_0 = 6.20$ for the higher fraction (with 95% CI of 6.16–6.23, $N = 6609$). The change was within 4%. The generation period has changed even less: from $T_{gen} = 6.88$ for the lower fraction (95% CI of 6.81–6.94, $N = 6318$), to $T_{gen} = 6.93$ (with 95% CI of 6.87–6.99, $N = 6609$). This change stayed within 1%, with confidence intervals overlapping. Such low sensitivity is in agreement with

our prior analysis showing slow linear increases of R_0 and T_{gen} in response to changes in the fraction σ_c (Chang et al., 2020).

Our Australia-wide modelling used the higher fraction: $\sigma_c = 0.268$, resulting in $R_0 = 6.20$ and $T_{gen} = 6.93$. In concordance with the NCIRS report, this setting resulted in the fraction of cases in children averaging $A_c = 0.22$ (computed for the period from 2 July to 19 August, with $SD = 0.5$), and varying in the range between 0.17 and 0.31, as shown in Fig. S6. The levels $SD = 0.4$ and $SD = 0.6$ produced similar, largely overlapping profiles (see Supplementary Data 1).

4 GROWTH RATES

To estimate growth rates β, we fit a 7-day moving average of the corresponding incidence time series $I(t)$ to an exponential function $\alpha \exp(\beta(t))$, using MATLAB R2020a function $movmean(I, [6\ 0])$. The growth rate of the observed incidence were estimated for several time periods (see Table S7).

The growth rates β_{SD} for the time series simulated for each SD level between 0.0 and 1.0 were estimated for the periods lasting from either the start of initial restrictions (27 June), or from 16 July (comprehensive lockdown measures were announced on 17 July).

5 HOSPITALISATIONS AND FATALITIES

In computing daily hospitalisations, as fractions of case incidence, we used age-dependent case hospitalisation risks (CHRs) reported for the Alpha (B.1.1.7) variant (Nyberg et al., 2021), and scaled up to the Delta variant. To scale the CHRs, we run a linear regression between (i) the hospitalisations computed according to the CHRs determined for the Alpha variant (Nyberg et al., 2021), and (ii) the actual hospitalisations reported in Australia between 16 June and 24 September 2021 (cov, 2021). The regression produced a good fit ($R^2 = 0.988$), with the multiplier of 1.715 and the additive constant of 43.38, see Fig. S7. The CHR estimates are shown in Table S8. Using the case incidence projections (for vaccinated and unvaccinated agents) and the CHRs, the hospitalisations were computed with time offset of 5 days (Health Protection NSW, 2021b), and the average hospital stay of 14 days. The vaccine efficacy against severe disease was assumed to be above 90% (specifically, VEh= 0.95) (Nasreen et al., 2021; Stowe et al., 2021), reducing the number of hospitalised vaccinated agents accordingly, before computing the resultant occupancy.

Age-dependent rate estimates for daily ICU admissions, as fractions of daily hospitalisations, were approximated by determining the corresponding ratios between the average ICU occupancy and the average hospitalisations (occupancy), reported in NSW between 16 June and 28 August 2021 (Health Protection NSW, 2021b). These estimates are included in Table S8. Using the hospitalisations (for vaccinated and unvaccinated agents) and the ICU admission rates, the ICU demand was computed with the average hospital stay of 18 days (Burrell et al., 2021).

To estimate potential fatalities, as fractions of case incidence, we use a meta-regression equation for age-dependent infection fatality rates (IFRs) (Levin et al., 2020):

$$\log_{10}(\text{IFR}) = -3.27 + 0.0524 \times \text{age} \tag{S7}$$

The IFR for the age over 80 years was truncated at the IFR for 80 years. Using the case incidence projections (for vaccinated and unvaccinated agents) and the IFRs, the fatalities were computed with time offset of 11 days (Health Protection NSW, 2021b). The vaccine efficacy against death was assumed to be above 90% (VEf= 0.95) (Nasreen et al., 2021; Stowe et al., 2021; Hyde et al., 2021), reducing the number of fatalities among vaccinated agents accordingly.

REFERENCES

Cliff OM, Harding M, Piraveen M, Erten Y, Gambhir M, Prokopenko M. Investigating spatiotemporal dynamics and synchrony of influenza epidemics in Australia: an agent-based modelling approach. *Simulation Modelling Practice and Theory* **87** (2018) 412–431.

Zachreson C, Fair K, Cliff OM, Harding M, Piraveenan M, Prokopenko M. Urbanization affects peak timing, prevalence, and bimodality of influenza pandemics in Australia: results of a census-calibrated model. *Science Advances* **4** (2018) eaau5294.

Fair KM, Zachreson C, Prokopenko M. Creating a surrogate commuter network from Australian Bureau of Statistics census data. *Scientific Data* **6** (2019) 150.

Chang SL, Harding N, Zachreson C, Cliff OM, Prokopenko M. Modelling transmission and control of the COVID-19 pandemic in Australia. *Nature Communications* **11** (2020) 1–13.

Zachreson C, Fair KM, Harding N, Prokopenko M. Interfering with influenza: nonlinear coupling of reactive and static mitigation strategies. *Journal of The Royal Society Interface* **17** (2020) 20190728.

Germann TC, Kadau K, Longini IM, Macken CA. Mitigation strategies for pandemic influenza in the United States. *Proceedings of the National Academy of Sciences* **103** (2006) 5935–5940.

Miller JC. Spread of infectious disease through clustered populations. *Journal of the Royal Society Interface* **6** (2009) 1121–1134.

Bernal JL, Andrews N, Gower C, Gallagher E, Simmons R, Thelwall S, et al. Effectiveness of COVID-19 vaccines against the B.1.617.2 variant. *medRxiv* (2021).

Zachreson C, Chang SL, Cliff OM, Prokopenko M. How will mass-vaccination change COVID-19 lockdown requirements in Australia? *The Lancet Regional Health – Western Pacific* **14** (2021) 100224.

Harris RJ, Hall JA, Zaidi A, Andrews NJ, Dunbar JK, Dabrera G. Impact of vaccination on household transmission of SARS-CoV-2 in England. *medRxiv* (2021).

Campbell F, Archer B, Laurenson-Schafer H, Jinnai Y, Konings F, Batra N, et al. Increased transmissibility and global spread of SARS-CoV-2 variants of concern as at June 2021. *Eurosurveillance* **26** (2021) 2100509.

Efron B, Tibshirani RJ. *An introduction to the bootstrap*, *Monographs on statistics and applied probability*, vol. 57 (Chapman and Hall New York) (1994), 1–436 .

Ferretti L, Wymant C, Kendall M, Zhao L, Nurtay A, Abeler-Dörner L, et al. Quantifying SARS-CoV-2 transmission suggests epidemic control with digital contact tracing. *Science* **368** (2020).

Wu JT, Leung K, Bushman M, Kishore N, Niehus R, de Salazar PM, et al. Estimating clinical severity of COVID-19 from the transmission dynamics in Wuhan, China. *Nature Medicine* **26** (2020) 506–510.

Zhang M, Xiao J, Deng A, Zhang Y, Zhuang Y, Hu T, et al. Transmission dynamics of an outbreak of the COVID-19 Delta variant B.1.617.2 – Guangdong Province, China, May–June 2021. *Chinese Center for Disease Control and Prevention* **3** (2021) 584–586.

[Dataset] Centers for Disease Control and Prevention. Interim Guidance on Duration of Isolation and Precautions for Adults with COVID-19. `https://www.cdc.gov/coronavirus/2019-ncov/hcp/duration-isolation.html` (2020).

Arons MM, Hatfield KM, Reddy SC, Kimball A, James A, Jacobs JR, et al. Presymptomatic SARS-CoV-2 infections and transmission in a skilled nursing facility. *New England Journal of Medicine* **382** (2020) 2081–2090.

Wölfel R, Corman VM, Guggemos W, Seilmaier M, Zange S, Müller MA, et al. Virological assessment of hospitalized patients with COVID-2019. *Nature* **581** (2020) 465–469.

Lauer SA, Grantz KH, Bi Q, Jones FK, Zheng Q, Meredith HR, et al. The incubation period of coronavirus disease 2019 (COVID-19) from publicly reported confirmed cases: estimation and application. *Annals*

of Internal Medicine **172** (2020) 577–582.

[Dataset] Danchin M, Koirala A, Russell F, Britton P. Is it more infectious? Is it spreading in schools? This is what we know about the Delta variant and kids. https://theconversation.com/is-it-more-infectious-is-it-spreading-in-schools-this-is-what-we-know-about-the-delta-variant-and-kids-163724 (2021). Accessed on 10 July 2021.

[Dataset] Health Protection NSW. COVID-19 (Coronavirus) statistics. 28 June 2021. https://www.health.nsw.gov.au/news/Pages/20210628_00.aspx (2021a). Accessed on 10 July 2021.

[Dataset] NCIRS. COVID-19 in schools and early childhood education and care services – the experience in NSW: 16 June to 31 July 2021. National Centre for Immunisation Research and Surveillance (NCIRS). https://www.ncirs.org.au/sites/default/files/2021-09/NCIRS%20NSW%20Schools%20COVID_Summary_8%20September%2021_Final.pdf (2021). Accessed on 9 September 2021.

Macartney K, Quinn HE, Pillsbury AJ, Koirala A, Deng L, Winkler N, et al. Transmission of SARS-CoV-2 in Australian educational settings: a prospective cohort study. *The Lancet Child & Adolescent Health* **4** (2020) 807–816.

Nyberg T, Twohig KA, Harris RJ, Seaman SR, Flannagan J, Allen H, et al. Risk of hospital admission for patients with SARS-CoV-2 variant B.1.1.7: cohort analysis. *BMJ* **373** (2021).

[Dataset] COVID-19 in Australia. https://www.covid19data.com.au/ (2021). Accessed on 15 November 2021.

[Dataset] Health Protection NSW. COVID-19 Weekly Surveillance in NSW. Epidemiological Week 34, Ending 28 August 2021. https://www.health.nsw.gov.au/Infectious/covid-19/Pages/weekly-reports.aspx (2021b). Accessed on 18 September 2021.

Nasreen S, He S, Chung H, Brown KA, Gubbay JB, Buchan SA, et al. Effectiveness of COVID-19 vaccines against variants of concern, Canada. *medRxiv* (2021).

Stowe J, Andrews N, Gower C, Gallagher E, Utsi L, Simmons R, et al. Effectiveness of COVID-19 vaccines against hospital admission with the Delta (B.1.617.2) variant. *medRxiv* (2021).

Burrell AJ, Pellegrini B, Salimi F, Begum H, Broadley T, Campbell LT, et al. Outcomes for patients with COVID-19 admitted to Australian intensive care units during the first four months of the pandemic. *Medical Journal of Australia* **214** (2021) 23–30.

Levin AT, Hanage WP, Owusu-Boaitey N, Cochran KB, Walsh SP, Meyerowitz-Katz G. Assessing the age specificity of infection fatality rates for COVID-19: systematic review, meta-analysis, and public policy implications. *European Journal of Epidemiology* (2020) 1–16.

Hyde Z, Parslow J, Grafton ARQ, Kompas AT. What vaccination coverage is required before public health measures can be relaxed in Australia? *OSF* (2021) osf.io/gs7zn/.

[Dataset] Australian Government, Department of Health. COVID-19 Vaccine Roll-out. https://www.health.gov.au/sites/default/files/documents/2021/<MM>/covid-19-vaccine-rollout-update-<DD>-<month>-2021.pdf (2021). Accessed on 1 July 2021, 17 July 2021, 17 August 2021, 16 September 2021, 17 October 2021.

[Dataset] Blanquart F, Abad C, Ambroise J, Bernard M, Cosentino G, Giannoli JM, et al. Spread of the Delta variant, vaccine effectiveness against PCR-detected infections and within-host viral load dynamics in the community in France. https://hal.archives-ouvertes.fr/hal-03289443/ (2021). Accessed on 1 August 2021.

6 SUPPLEMENTARY TABLES AND FIGURES

Table S1. Daily transmission rates $q^g_{j\to i}$ for different contact groups g. The age is assigned an integer value.

Contact Group g	Infected Individual j	Susceptible Individual i	Transmission Probability $q^g_{j\to i}$
Household size 2	Any	Child ($\leq$ 18)	0.0933
	Any	Adult ($\geq$ 19)	0.0242
Household size 3	Any	Child ($\leq$ 18)	0.0586
	Any	Adult ($\geq$ 19)	0.0149
Household size 4	Any	Child ($\leq$ 18)	0.0417
	Any	Adult ($\geq$ 19)	0.0106
Household size 5	Any	Child ($\leq$ 18)	0.0321
	Any	Adult ($\geq$ 19)	0.0081
Household size 6	Any	Child ($\leq$ 18)	0.0259
	Any	Adult ($\geq$ 19)	0.0065
School	Child ($\leq$ 18)	Child ($\leq$ 18)	0.00029
Grade	Child ($\leq$ 18)	Child ($\leq$ 18)	0.00158
Class	Child ($\leq$ 18)	Child ($\leq$ 18)	0.00865

Table S2. Daily contact rates $c^g_{j\to i}$ for different contact groups g. The age is assigned an integer value.

Mixing group g	Infected individual j	Susceptible individual i	Contact probability $c^g_{j\to i}$
Household cluster	Child ($\leq$ 18)	Child ($\leq$ 18)	0.05
	Child ($\leq$ 18)	Adult ($\geq$ 19)	0.05
	Adult ($\geq$ 19)	Child ($\leq$ 18)	0.05
	Adult ($\geq$ 19)	Adult ($\geq$ 19)	0.05
Working Group	Adult (19-64)	Adult (19-64)	0.05
Neighbourhood	Any	Child (0-4)	0.0000435
	Any	Child (5-18)	0.0001305
	Any	Adult (19-64)	0.000348
	Any	Adult ($\geq$ 65)	0.000696
Community	Any	Child (0-4)	0.0000109
	Any	Child (5-18)	0.0000326
	Any	Adult (19-64)	0.000087
	Any	Adult ($\geq$ 65)	0.000174

Table S3. Main parameters for AMTraC-19 transmission model.

parameter	value	distribution	notes
κ	5.3	NA	global transmission scalar
T_{inc}	4.4 days (mean)	lognormal ($\mu = 1.396$, $\sigma = 0.413$)	incubation period
T_{rec}	10.5 days (mean)	uniform [7, 14] days	symptomatic (or asymptomatic) period
α	0.5	NA	asymptomatic transmission scalar
ρ	0.08	NA	contact-to-transmission scalar
σ_a	0.67	NA	probability of symptoms in adults (age > 18)
σ_c	0.134 or 0.268	NA	probability of symptoms in children (age ≤ 18)
r_{symp}	0.227	NA	daily case detection rate (symptomatic)
r_{asymp}	0.01	NA	daily case detection rate (asymptomatic)

Table S4. The macro-distancing parameters and interaction strengths of NPIs. An example simulation scenario set for 28 weeks (196 days), with SD and SC synchronised to be triggered by 400 cumulative cases and last for 121 days.

	Macro-distancing			Micro-distancing (interaction strengths)			
Intervention	Compliance level	Duration T	Threshold	Household	Community	Workplace/School	Duration t
CI	0.7	196	0	1.0	0.25	0.25	$D(i)$
HQ	0.5	196	0	2.0	0.25	0.25	14
SC^c	1.0	121	400	1.0	0.5	0	121
SC^a	0.5	121	400	1.0	0.5	0	121
SD	0.5	121	400	1.0	0.25	0.1	121

Table S5. Simulated and actual (Australian Government, Department of Health, 2021) vaccination coverage across Australia (double vaccinated individuals).

date	Adults (16+): actual (%)	Adults (16+): simulated (%)	Total population: simulated (%)
16 July	13.35	17.28	13.82
16 August	26.88	33.51	26.80
15 September	44.66	49.21	39.36
16 October	67.85	65.44	52.35
13 November	83.01	79.38	63.49

Table S6. Calibration targets.

parameter	value from ABM [range]	sample size	target value [range]	notes
R_0 ($\sigma_c = 0.134$)	5.97 [5.93, 6.00]	6318	[5.5, 6.5]	basic reproductive ratio (Campbell et al., 2021)
T_{gen} ($\sigma_c = 0.134$)	6.88 [6.81, 6.94]	6318	[5.8, 8.1]	generation/serial interval (Campbell et al., 2021) (Wu et al., 2020; Blanquart et al., 2021)
R_0 ($\sigma_c = 0.268$)	6.20 [6.16, 6.23]	6609	[5.5, 6.5]	basic reproductive ratio (Campbell et al., 2021)
T_{gen} ($\sigma_c = 0.268$)	6.93 [6.87, 6.99]	6609	[5.8, 8.1]	generation/serial interval (Campbell et al., 2021) (Wu et al., 2020; Blanquart et al., 2021)
β_I	$\beta_{0.4} = 0.084$	10	0.098 [0.084, 0.112]	growth rate, case incidence (NSW: 17 June–13 July)
β_{III}	$\beta_{0.6} = 0.029$	10	0.037 [0.026, 0.048]	growth rate, case incidence (NSW: 16–25 July)
A_c ($\sigma_c = 0.268$)	A_c ($SD = 0.5$) = 0.22 [0.16, 0.29]	10	0.27 [0.22, 0.29]	fraction of cases in children (age ≤ 18) (NSW: 16 June–19 August) (NCIRS, 2021)

Table S7. The growth rate of the observed incidence.

growth rate	period	mean	95% CI
β_I	17 June – 13 July	0.098	[0.084, 0.112]
β_{II}	17 June – 25 July	0.076	[0.069, 0.084]
β_{III}	16 July – 25 July	0.037	[0.026, 0.048]

Table S8. Estimates of age-dependent case hospitalisation risks (CHRs, %) and ICU admission rates.

Rate \Age	0-9	10-19	20-29	30-39	40-49	50-59	60-69	70-79	80+
CHR (Alpha) (Nyberg et al., 2021)	0.9	0.7	1.9	3.4	5.0	7.2	10.6	16.9	21.7
CHR (Delta)	1.54	1.20	3.26	5.83	8.58	12.35	18.18	28.98	37.22
ICU (Delta)	0.01	0.06	0.09	0.9	0.13	0.19	0.19	0.17	0.09

Table S9. Estimates (across Australia) of the peak demand in hospitalisations and ICUs, for various vaccine efficacies (VEi= 0.5 is used in primary analysis, varied to VEi= 0.35 and VEi= 0.65 in sensitivity analysis).

	Peak hospitalisations: mean and 95% CI			Peak ICU demand: mean and 95% CI		
Scenario	Total	Vaccinated	Unvaccinated	Total	Vaccinated	Unvaccinated
VEi = 0.5						
$SD = 0.4$	4805 [4282, 5257]	113 [102, 122]	4706 [4221, 5154]	812 [731, 885]	18 [16, 20]	795 [711, 868]
$SD = 0.5$	1604 [1358, 1844]	33 [29, 38]	1575 [1331, 1809]	272 [230, 312]	5.7 [4.8, 6.5]	267 [227, 306]
$SD = 0.6$	533 [476, 579]	8.7 [7.7, 9.3]	526 [467, 571]	91 [80, 99]	1.5 [1.3, 1.6]	89 [79, 97]
VEi = 0.35						
$SD = 0.4$	7216 [6460, 7771]	158 [176, 188]	7061 [6296, 7616]	1225 [1093, 1314]	28 [25, 31]	1197 [1069, 1284]
$SD = 0.5$	2249 [1882, 2657]	45 [53, 61]	2204 [1851, 2600]	381 [320, 449]	8.6 [7.2, 10]	374 [314, 441]
$SD = 0.6$	620 [571, 668]	10.9 [11.8, 12.5]	610 [562, 658]	105 [97, 114]	2 [1.9, 2.2]	103 [95, 111]
VEi = 0.65						
$SD = 0.4$	3817 [3497, 4068]	69.6 [63, 75]	3747 [3428, 3990]	650 [598, 692]	14.1 [13, 15]	637 [589, 681]
$SD = 0.5$	1252 [1172, 1336]	22 [21, 24]	1230 [1148, 1313]	213 [200, 228]	4.1 [3.8, 4.4]	210 [195, 224]
$SD = 0.6$	467 [414, 525]	6.9 [6.1, 7.7]	462 [411, 522]	79 [70, 89]	1.2 [1, 1.4]	78 [70, 88]

Table S10. Estimates (across Australia) of cumulative fatalities (15 October 2021), for various vaccine efficacies (VEi= 0.5 is used in primary analysis, varied to VEi= 0.35 and VEi= 0.65 in sensitivity analysis).

	Cumulative fatalities: mean and 95% CI		
Scenario	Total	Vaccinated	Unvaccinated
VEi = 0.5			
$SD = 0.4$	1201 [1057, 1326]	10 [8.8, 11.2]	1191 [1047, 1313]
$SD = 0.5$	539 [479, 624]	4.2 [3.5, 4.9]	535 [447, 615]
$SD = 0.6$	235 [209, 256]	1.7 [1.5, 1.8]	233 [205, 253]
VEi = 0.35			
$SD = 0.4$	1672 [1455, 1834]	14.3 [12.3, 15.7]	1658 [1435, 1817]
$SD = 0.5$	667 [553, 811]	5.4 [4.5, 6.5]	661 [543, 798]
$SD = 0.6$	259 [238, 280]	1.9 [1.8, 2.1]	257 [237, 278]
VEi = 0.65			
$SD = 0.4$	1094 [997, 1175]	8.9 [8.1, 9.7]	1082 [987, 1165]
$SD = 0.5$	461 [429, 494]	3.6 [3.4, 3.8]	458 [426, 490]
$SD = 0.6$	216 [191, 244]	1.5 [1.3, 1.7]	214 [189, 241]

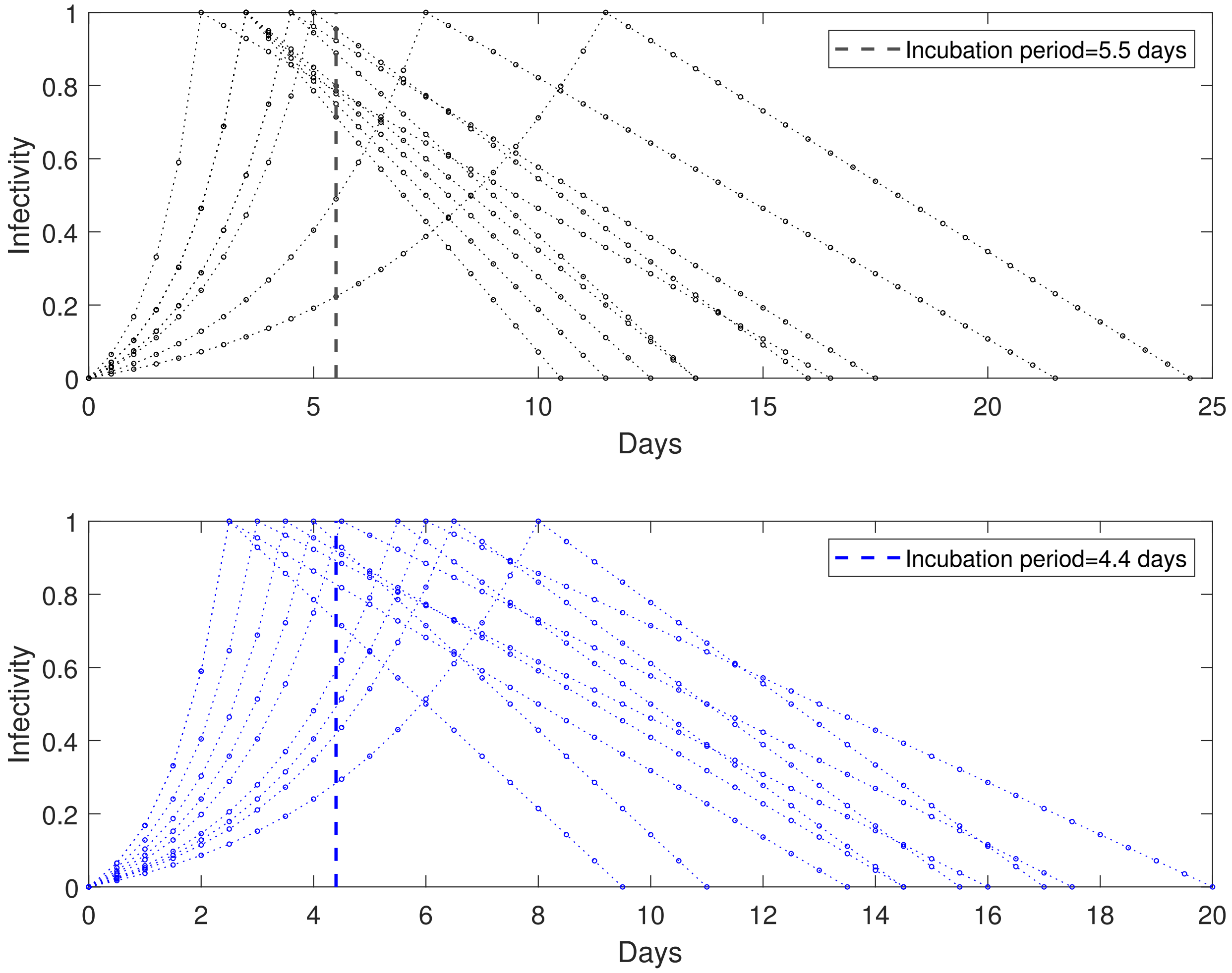

Figure S1. Model of the natural history of COVID-19. Profiles of the infectivity are sampled from 20 random agents, with each profile rising exponentially until a peak, followed by a linear decrease to full recovery. Vertical dashed lines indicate the mean incubation period T_{inc}: 5.5 days (top) and 4.4 days (bottom), with the means distributed log-normally.

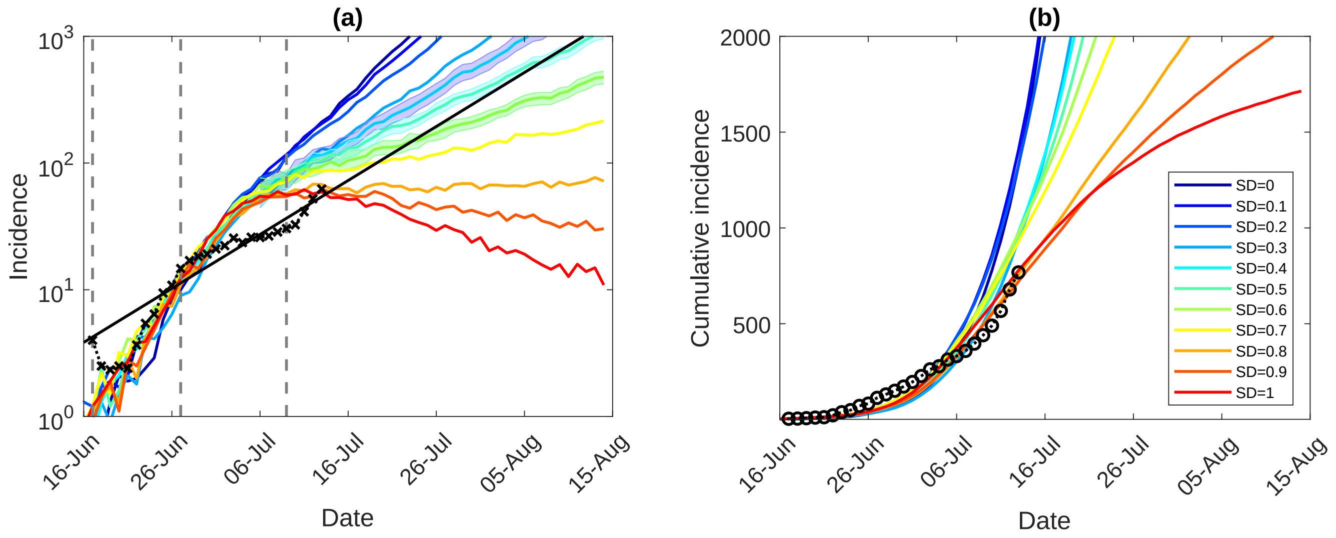

Figure S2. Sensitivity analysis for early interventions, moderate restrictions and shorter incubation period (NSW; progressive vaccination rollout; suppression threshold: 100 cases; $T_{inc} = 4.4$**,** $R_0 = 5.97$**,** $T_{gen} = 6.88$**)**: a comparison between simulation scenarios and actual epidemic curves up to 13 July, under moderate interaction strengths ($CI_c = CI_w = 0.25$, $HQ_c = HQ_w = 0.25$, $SD_c = 0.25$, $SC = 0.5$). A moving average of the actual time series for (a) (log-scale) incidence (crosses), and (b) cumulative incidence (circles); with an exponential fit of the incidence's moving average (black solid line showing $\beta_I = 0.098$). Vertical dashed marks align the simulated days with the outbreak start (17 June, day 11), initial restrictions (27 June, day 21), and tighter lockdown (9 July, day 33). Traces corresponding to each social distancing (SD) compliance level are shown as average over 10 runs (coloured profiles for SD varying in increments of 10%, i.e., between $SD = 0.0$ and $SD = 1.0$). 95% confidence intervals for incidence profiles, for $SD \in \{0.4, 0.5, 0.6\}$, are shown as shaded areas. Each SD intervention, coupled with school closures, begins with the start of initial restrictions, when cumulative incidence exceeds 100 cases (b: inset). The alignment between simulated days and actual dates may slightly differ across separate runs. Case isolation and home quarantine are in place from the outset.

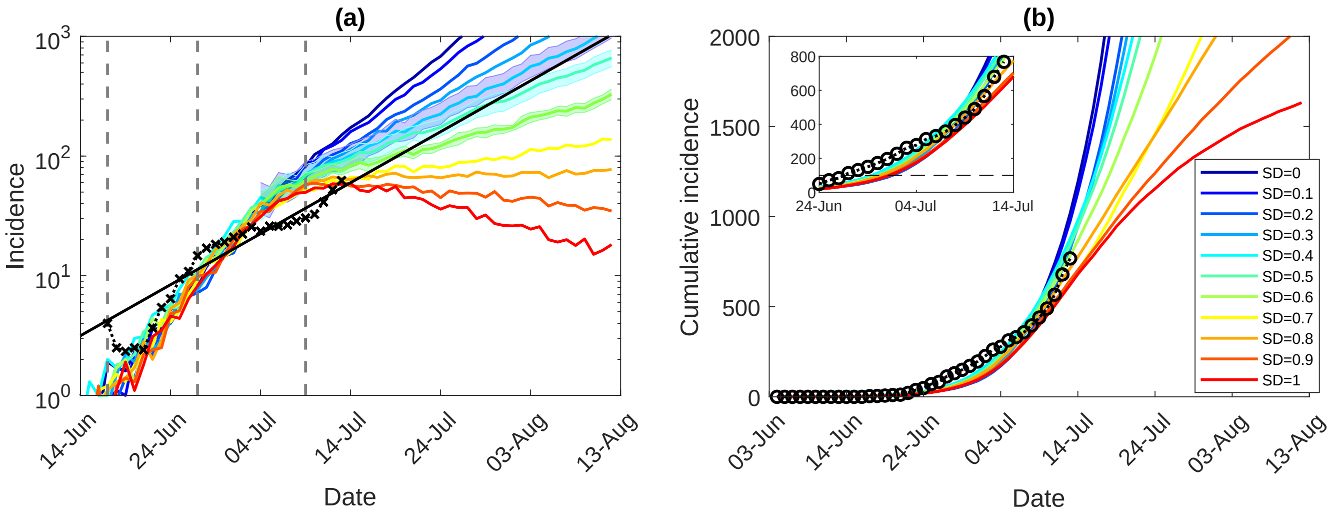

Figure S3. Sensitivity analysis for early interventions, moderate restrictions and longer incubation period (NSW; pre-pandemic vaccination rollout; suppression threshold: 100 cases; $T_{inc} = 5.5$, $R_0 = 6.09$, $T_{gen} = 7.74$): a comparison between simulation scenarios and actual epidemic curves up to 13 July, under moderate interaction strengths ($CI_c = CI_w = 0.25$, $HQ_c = HQ_w = 0.25$, $SD_c = 0.25$, $SC = 0.5$). A moving average of the actual time series for (a) (log-scale) incidence (crosses), and (b) cumulative incidence (circles); with an exponential fit of the incidence's moving average (black solid line showing $\beta_I = 0.098$). Vertical dashed marks align the simulated days with the outbreak start (17 June, day 13), initial restrictions (27 June, day 23), and tighter lockdown (9 July, day 35). Traces corresponding to each social distancing (SD) compliance level are shown as average over 10 runs (coloured profiles for SD varying in increments of 10%, i.e., between $SD = 0.0$ and $SD = 1.0$). 95% confidence intervals for incidence profiles, for $SD \in \{0.4, 0.5, 0.6\}$, are shown as shaded areas. Each SD intervention, coupled with school closures, begins with the start of initial restrictions, when cumulative incidence exceeds 100 cases (b: inset). The alignment between simulated days and actual dates may slightly differ across separate runs. Case isolation and home quarantine are in place from the outset.

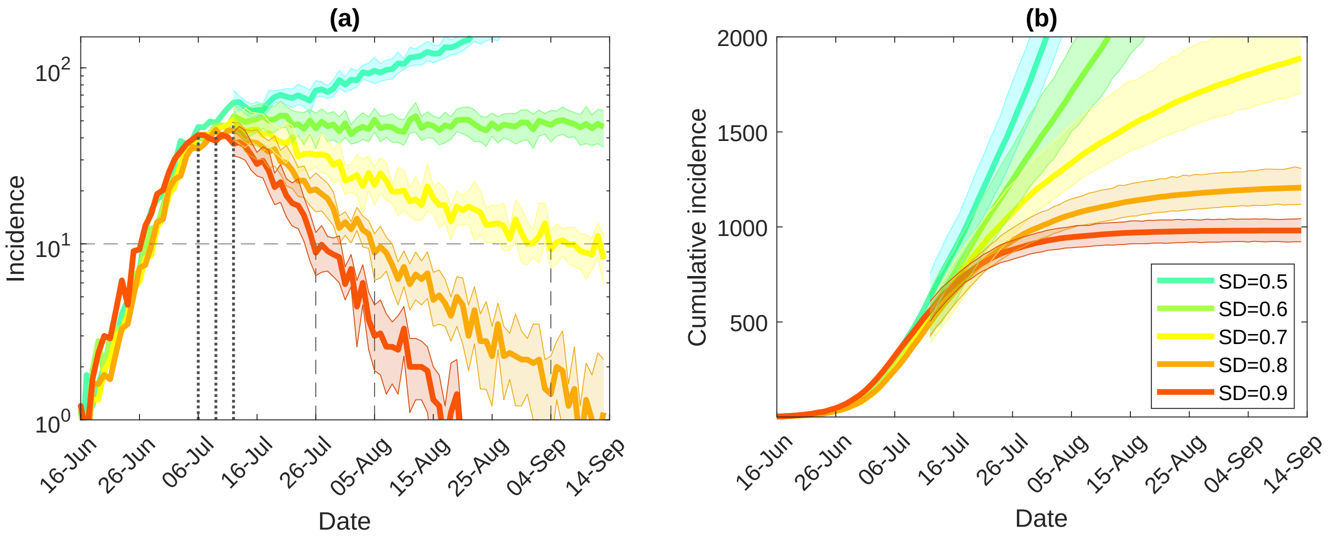

Figure S4. Sensitivity analysis for early interventions, tight restrictions and shorter incubation period (NSW; pre-pandemic vaccination rollout; suppression threshold: 100 cases; $T_{inc} = 4.4$, $R_0 = 5.97$, $T_{gen} = 6.88$): counter-factual simulation scenarios, under lowest feasible interaction strengths ($CI_c = CI_w = 0.1$, $HQ_c = HQ_w = 0.1$, $SD_c = 0.1$, $SC = 0.1$), for (a) (log scale) incidence (crosses), and (b) cumulative incidence (circles). Traces corresponding to feasible social distancing (SD) compliance level are shown as average over 10 runs (coloured profiles for SD varying in increments of 10%, i.e., between $SD = 0.5$ and $SD = 0.9$). Vertical lines mark the incidence peaks (dotted) and reductions below 10 daily cases (dashed). 95% confidence intervals are shown as shaded areas. Each SD intervention, coupled with school closures, begins with the start of initial restriction, when cumulative incidence exceeds 100 cases (i.e., simulated day 21). The alignment between simulated days and actual dates may slightly differ across separate runs. Case isolation and home quarantine are in place from the outset.

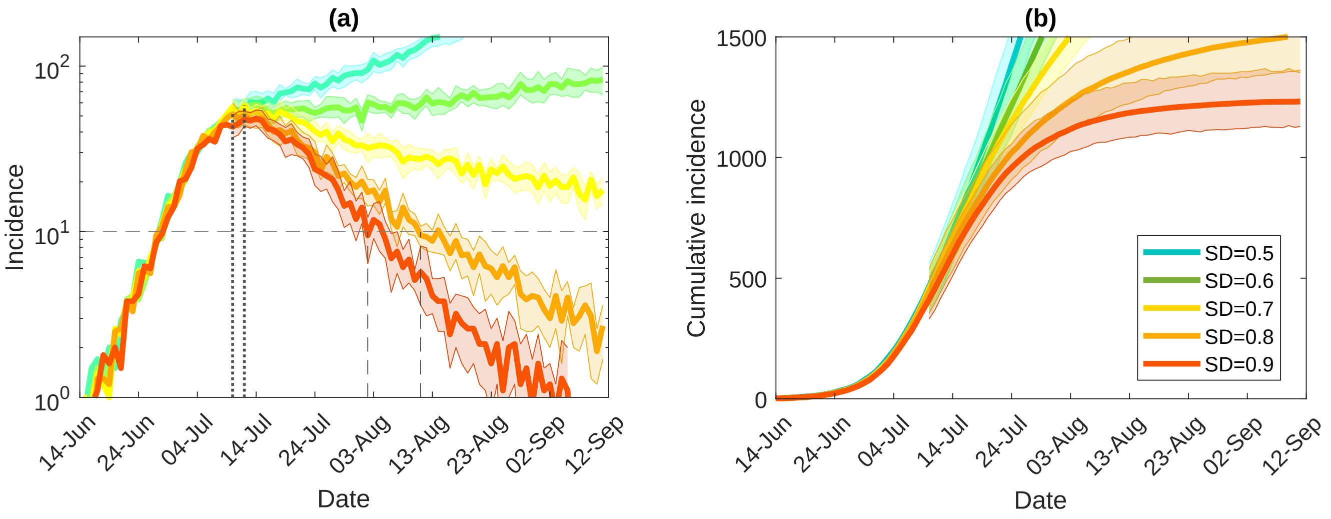

Figure S5. Sensitivity analysis for early interventions, tight restrictions and longer incubation period (NSW; pre-pandemic vaccination rollout; suppression threshold: 100 cases; $T_{inc} = 5.5$**,** $R_0 = 6.09$**,** $T_{gen} = 7.74$**)**: counter-factual simulation scenarios, under lowest feasible interaction strengths ($CI_c = CI_w = 0.1$, $HQ_c = HQ_w = 0.1$, $SD_c = 0.1$, $SC = 0.1$), for (a) (log scale) incidence (crosses), and (b) cumulative incidence (circles). Traces corresponding to feasible social distancing (SD) compliance level are shown as average over 10 runs (coloured profiles for SD varying in increments of 10%, i.e., between $SD = 0.5$ and $SD = 0.9$). 95% confidence intervals are shown as shaded areas. Each SD intervention, coupled with school closures, begins with the start of initial restrictions, when cumulative incidence exceeds 100 cases (i.e., simulated day 23). The alignment between simulated days and actual dates may slightly differ across separate runs. Case isolation and home quarantine are in place from the outset.

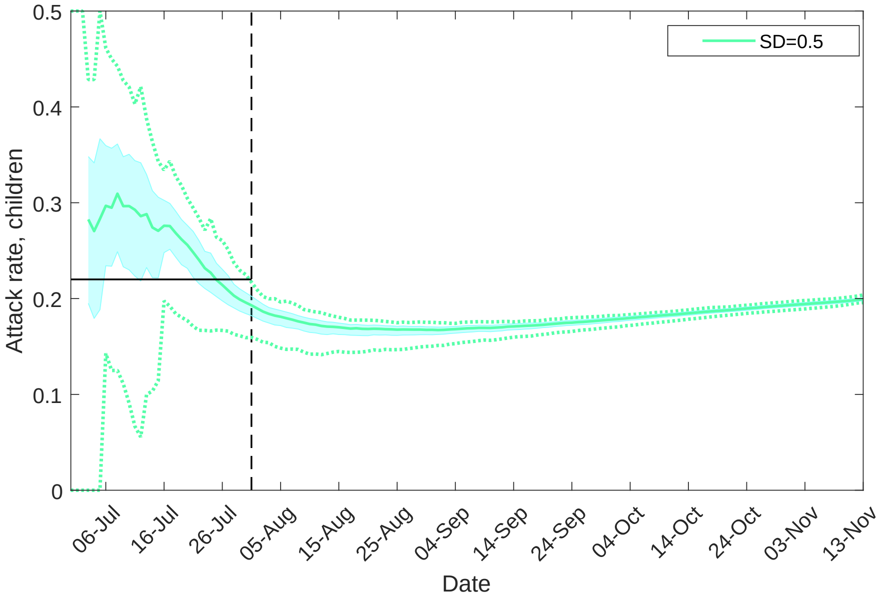

Figure S6. Fraction of cases in children across Australia. A trace corresponding to social distancing level $SD = 0.5$ is shown for the period between 30 June and 13 November as average over 10 runs. 95% confidence interval is shown as a shaded area. Minimal and maximal traces, per time point, are shown with dotted lines. The horizontal line shows the fraction of cases in children averaged for the period up to 31 July ($A_c = 0.22$), in agreement with the rate reported for the period between 16 June and 31 July (NCIRS, 2021). The SD intervention, coupled with school closures, begins with the start of initial restrictions. The alignment between simulated days and actual dates may slightly differ across separate runs. Case isolation and home quarantine are in place from the outset.

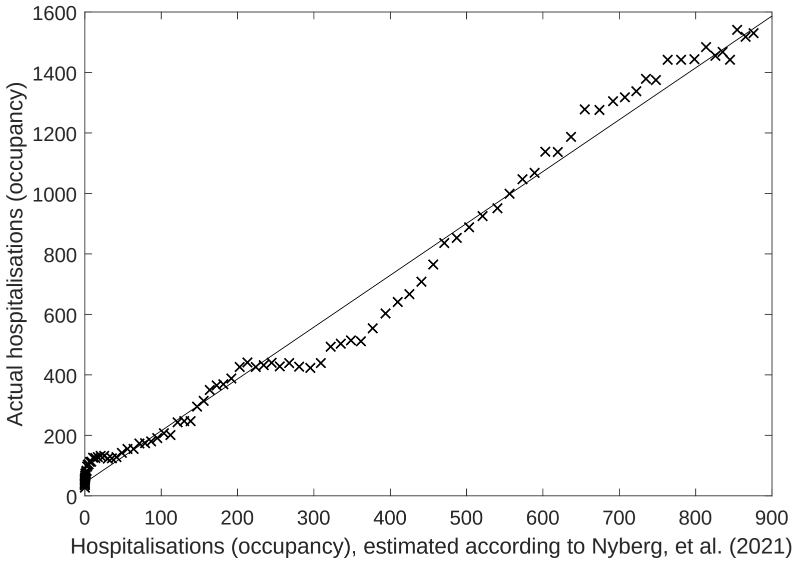

Figure S7. Scaling case hospitalisation risks (CHRs). A linear regression ($R^2 = 0.988$) between (i) the hospitalisations computed according to the CHRs determined for the Alpha variant (Nyberg et al., 2021), and (ii) the actual hospitalisations reported in Australia (cov, 2021) between 16 June and 24 September 2021.

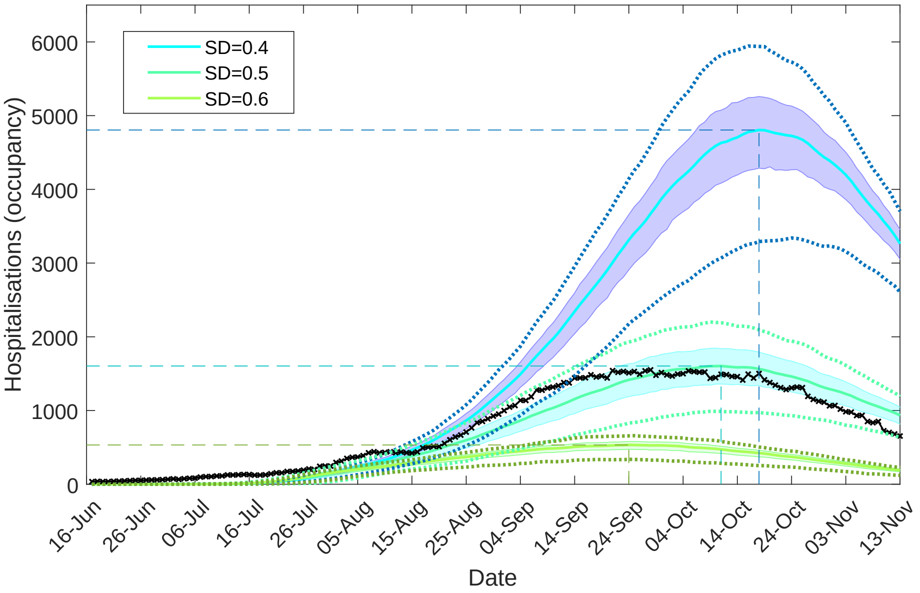

Figure S8. Estimated hospitalisations (occupancy) across Australia. Traces corresponding to social distancing levels $SD \in \{0.4, 0.5, 0.6\}$ are shown for the period between 16 June and 13 November, as averages over 10 runs (coloured profiles). 95% confidence intervals are shown as shaded areas. For each SD level, minimal and maximal traces, per time point, are shown with dotted lines. A moving average of the actual time series is shown in black. Peaks formed during the suppression period for each SD profile are identified with coloured dashed lines. The SD intervention, coupled with school closures, begins with the start of initial restrictions. The alignment between simulated days and actual dates may slightly differ across separate runs. Case isolation and home quarantine are in place from the outset.

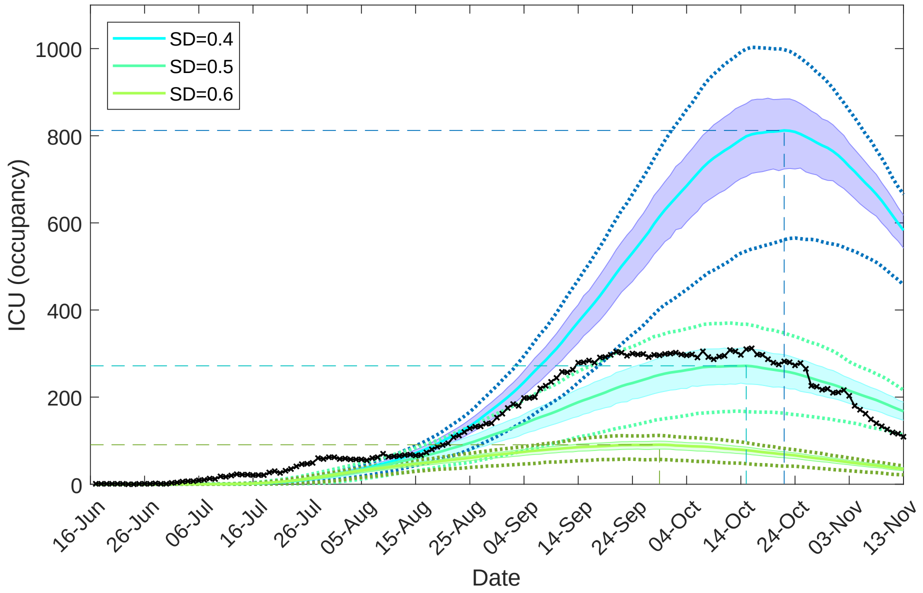

Figure S9. Estimated ICU demand across Australia. Traces corresponding to social distancing levels $SD \in \{0.4, 0.5, 0.6\}$ are shown for the period between 16 June and 13 November, as averages over 10 runs (coloured profiles). 95% confidence intervals are shown as shaded areas. For each SD level, minimal and maximal traces, per time point, are shown with dotted lines. A moving average of the actual time series is shown in black. Peaks formed during the suppression period for each SD profile are identified with coloured dashed lines. The SD intervention, coupled with school closures, begins with the start of initial restrictions. The alignment between simulated days and actual dates may slightly differ across separate runs. Case isolation and home quarantine are in place from the outset.

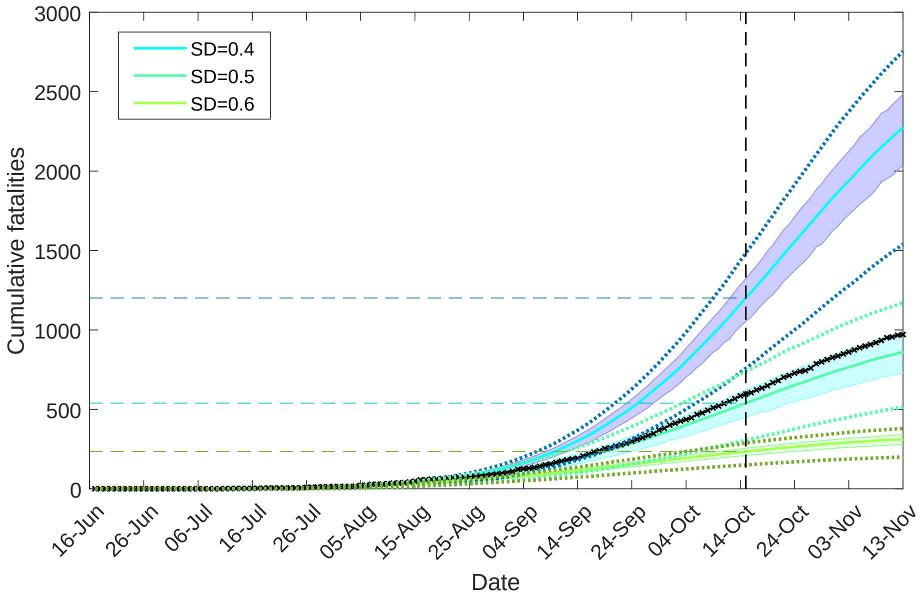

Figure S10. Estimated cumulative fatalities across Australia. Traces corresponding to social distancing levels $SD \in \{0.4, 0.5, 0.6\}$ are shown for the period between 16 June and 13 November, as averages over 10 runs (coloured profiles). 95% confidence intervals are shown as shaded areas. For each SD level, minimal and maximal traces, per time point, are shown with dotted lines. A moving average of the actual time series is shown in black. Dashed lines mark cumulative fatalities estimated for 15 October 2021, as reported in Table 3. The SD intervention, coupled with school closures, begins with the start of initial restrictions. The alignment between simulated days and actual dates may slightly differ across separate runs. Case isolation and home quarantine are in place from the outset.

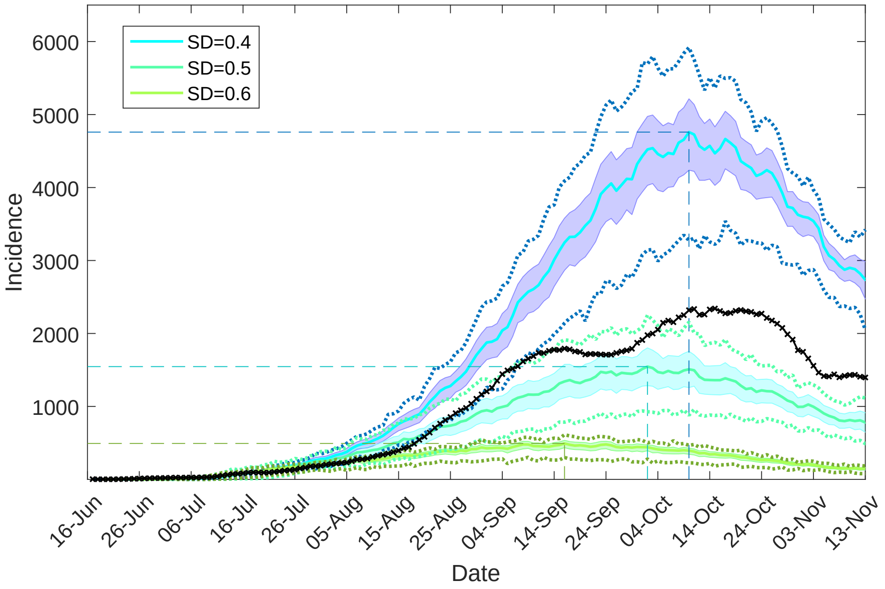

Figure S11. Moderate restrictions (Australia; progressive vaccination rollout; suppression threshold: 400 cases): a comparison between simulation scenarios and actual epidemic curves up to November 13, under moderate interaction strengths ($CI_c = CI_w = 0.25$, $HQ_c = HQ_w = 0.25$, $SD_c = 0.25$, $SC = 0.5$). A moving average of the actual time series for incidence is shown with crosses. Traces corresponding to social distancing levels $SD \in \{0.4, 0.5, 0.6\}$ are shown for the period between 16 June and 13 November, as averages over 10 runs (coloured profiles). 95% confidence intervals are shown as shaded areas. For each SD level, minimal and maximal traces, per time point, are shown with dotted lines. Peaks formed during the suppression period for each SD profile are identified with coloured dashed lines. Each SD intervention, coupled with school closures, begins with the start of initial restrictions. The alignment between simulated days and actual dates may slightly differ across separate runs. Case isolation and home quarantine are in place from the outset. Compare with Fig. 3.a showing the same data and simulated scenarios on a log scale.

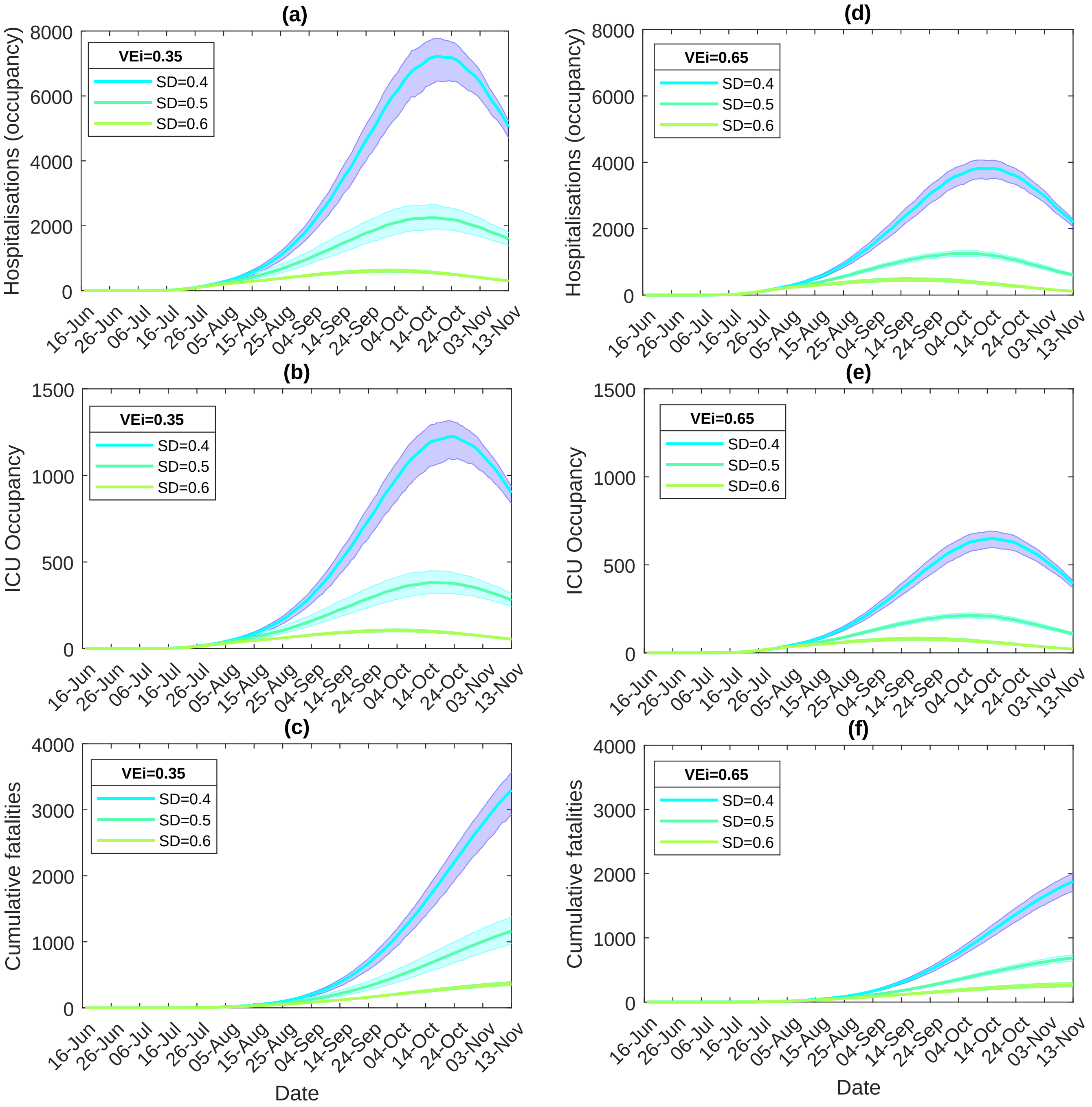

Figure S12. Estimated hospitalisations (occupancy), ICU demand, and cumulative fatalities across Australia; sensitivity analysis for different efficacies against infectiousness: VEi= 0.35, shown in left panels (a), (b), (c), and VEi= 0.65, shown in right panels (d), (e), (f). Traces corresponding to social distancing levels $SD \in \{0.4, 0.5, 0.6\}$ are shown for the period between 16 June and 13 November, as averages over 10 runs (coloured profiles). 95% confidence intervals are shown as shaded areas. The SD intervention, coupled with school closures, begins with the start of initial restrictions. The alignment between simulated days and actual dates may slightly differ across separate runs. Case isolation and home quarantine are in place from the outset.
